# ISGylation drives basal breast tumour progression by promoting EGFR recycling and Akt signalling

**DOI:** 10.1038/s41388-021-02017-8

**Published:** 2021-09-23

**Authors:** Alfonso Bolado-Carrancio, Martin Lee, Ailith Ewing, Morwenna Muir, Kenneth G. Macleod, William M. Gallagher, Lan K. Nguyen, Neil O. Carragher, Colin A. Semple, Valerie G. Brunton, Patrick T. Caswell, Alex von Kriegsheim

**Affiliations:** 1 Edinburgh Cancer Research Centre, Institute of Genetics and Cancer, University of Edinburgh, Crewe Road South, Edinburgh, EH4 2XU UK; 2 MRC Human Genetics Unit, Institute of Genetics and Cancer, University of Edinburgh, Crewe Road South, Edinburgh, EH4 2XU UK; 3 UCD School of Biomolecular and Biomedical Science, UCD Conway Institute, University College Dublin, Dublin, D4 Republic of Ireland; 4 Department of Biochemistry and Molecular Biology, Biomedicine Discovery Institute, Monash University, Melbourne, Victoria 3800 Australia; 5 Wellcome Trust Centre for Cell-Matrix Research, School of Biological Sciences, Faculty of Biology Medicine and Health, Manchester Academic Health Science Centre, The University of Manchester, Manchester, UK

**Keywords:** Proteomics, Breast cancer, Cell signalling

## Abstract

ISG15 is an ubiquitin-like modifier that is associated with reduced survival rates in breast cancer patients. The mechanism by which ISG15 achieves this however remains elusive. We demonstrate that modification of Rab GDP-Dissociation Inhibitor Beta (GDI2) by ISG15 (ISGylation) alters endocytic recycling of the EGF receptor (EGFR) in non-interferon stimulated cells using CRISPR-knock out models for ISGylation. By regulating EGFR trafficking, ISGylation enhances EGFR recycling and sustains Akt-signalling. We further show that Akt signalling positively correlates with levels of ISG15 and its E2-ligase in basal breast cancer cohorts, confirming the link between ISGylation and Akt signalling in human tumours. Persistent and enhanced Akt activation explains the more aggressive tumour behaviour observed in human breast cancers. We show that ISGylation can act as a driver of tumour progression rather than merely being a bystander.

## Introduction

Interferon-Induced 15 kDa protein (ISG15) was the first ubiquitin-like protein identified. However, its research was long restricted to the field of immune response where it was initially discovered. Recently, ISG15 has been associated with processes and pathologies distinct from the innate-immune response [1]. Tumour progression and aggressiveness of several cancer types, including endometrium, bladder, prostate, melanoma, colorectal, liver and breast cancer [2–9] has been correlated to ISG15 expression. However, the mechanism through which ISG15 regulates tumorigenesis remains elusive.

ISG15, like other ubiquitin-like proteins, can be covalently bound to lysine residues of target proteins [10] in a process known as ISGylation. This post-translational modification is similar to ubiquitination, as it requires a cascade involving three different ligases. The process can be reversed by the action of Ubiquitin Specific Peptidase 18 (USP18), a member of the deubiquitinase family which is the only ISG15-specific deubiquitinase enzyme that has been described so far [11].

Basal levels of ISG15 and ISGylation-related enzymes are generally low in cells. Protein expression can be substantially increased by a variety of stimuli, such as type I interferons [12], Lipopolysaccharide [13], growth factors [14] or viral infections [15]. In breast cancer cells, enhanced ISG15 expression is induced through exosome-mediated cGAS activation [9, 16] and from nuclear DNA release after DNA damage [17].

ISG15 functions either as unconjugated or as a covalently linked protein. When released into the extracellular matrix [18], it functions as an immunomodulatory agent for lymphocytes [19] or Natural Killer (NK) cells [20]. The role of secreted ISG15 during tumour development is contradictory. It has been described both as an antitumoural factor by increasing NK cell infiltration in xenografts [21] or tumourigenic by increasing the invasive potential of primary tumour cells [22]. As a conjugate, ISG15 is widely linked to immune functions. Initially it was considered an antiviral protein [23], although recent data in human models suggest that ISG15 functions as a negative effector of type I interferon signalling rather than directly regulating the immune system [24].

To comprehensively map the role of ISGylation numerous mass spectrometry-based studies have been carried out to identify substrates of ISG15 modification, the ISGylome [25–27]. These studies showed that ISG15 substrates are associated with multiple signalling pathways and are cell/tissue type dependent, thus suggesting that ISG15 might play beyond the interferon-associated response. However, only a few of these putative targets have been validated endogenously and even fewer have been functionally characterised by identifying the molecular role of the ISGylation. Despite this, it is apparent that the molecular function of ISGylation is varied. It has been shown to induce both protein stabilisation [28] and degradation [29, 30], as well as modulating protein–protein interactions [31, 32]. This plasticity makes ISG15 a dynamic post-translation modification that can regulate substrate function disparately.

In breast cancer models, ISG15 and/or ISGylation correlate with aggressive features such as, cell cycle progression, cell motility and tumour growth in xenograft models [28, 29, 33], yet, we still do not know if ISG15 is just a bystander or indeed a driver. Progress in uncovering a functional link has been hampered by the lack of functional, mechanistic insight into how ISGylation regulates signalling networks. We decided to bridge this gap by applying unbiased, systems approaches to elucidate in detail how ISGylation contributes to cell signalling.

In this study, we aimed to identify the molecular mechanisms that explains why high ISG15/ISGylation correlates with poor patient prognosis in breast cancer.

## Results

### ISGylation negatively correlates with disease-free survival

**Fig. 1:**
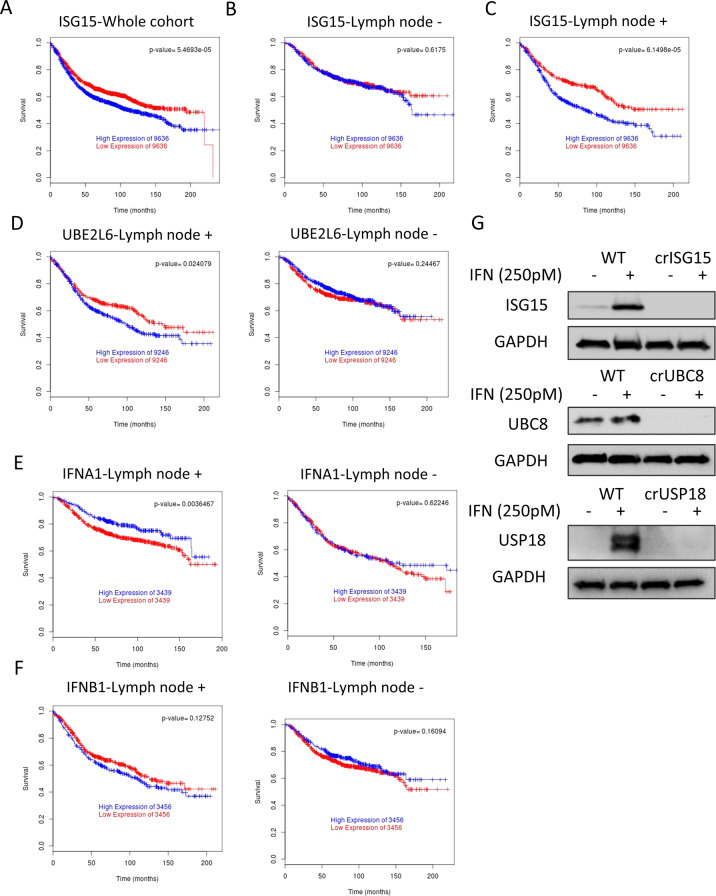
ISG15 expression is associated with poor outcome in breast cancer patients with lymph-node metastasis. **A** Kaplan–Meier plot of disease-free survival of breast cancer patients (*n* = 2643) separated into two groups with high (blue) or low (red) mRNA expression levels of ISG15. **B** Kaplan-Meier plot of free disease survival of patients from A that are negative for tumour cells in lymph nodes (*n* = 740). **C** Kaplan-Meier plot of free disease survival of patients from A positive for tumour presence in lymph nodes (*n* = 1178). **D** Kaplan-Meier plot of disease-free survival association with mRNA levels of UBE2L6, separated into two groups with high (blue) or low (red) levels, in lymph node positive patients (*n* = 744) or lymph node negative patients (*n* = 350). As in **D** analysis of mRNA levels in lymph node positive and negative of the interferon coding genes IFNA1 (*n* = 600) (**E**) and IFNB1 (*n* = 744) (**F**). **G** WB analysis of the indicated representative clones to confirm the knockout, cells were treated for 48 h with either vehicle or IFNb1a 250 pM to boost the protein levels of the different ISGylation-related proteins.

We used the Breastmark database [34] to analyse the correlation between prognosis, ISG15 levels and metastasis. As previously shown, elevated ISG15 mRNA levels correlated with lower disease-free survival (Fig. 1A). Surprisingly however, the correlation was only maintained in patients with identified lymph node metastasis (Fig. 1B, C).

ISG15 can function as a conjugated or free/secreted protein, to narrow down which form is associated with this correlation, we determined if regulators of ISGylation showed an analogous correlation. We correlated disease free-survival with genes encoding the ISG15 E1 ligase UBE1L, (UBA7) [35], and the main ISG15 E2 ligase UBC8/UbCH8 (UBE2L6) [36]. As seen with ISG15, UBE2L6 mRNA expression levels negatively correlated with survival in patients with cancer that had spread to the lymph nodes whereas the correlation was lost in patients without lymph node metastasis (Fig. 1D). However, this correlation is not detected for UBEL1 (Fig. S1A). Furthermore, the analysis of a second ISG15 E2 ligase, UBCh6 (UBE2E1) [37] did not show a correlation with survival either (Fig. S1B). These data lead us to hypothesise that the correlation between ISG15 expression and survival was not because of a global increase in ISGylation, but was rather due to the specific, UBC8-dependent ISGylation of a subgroup of proteins.

Both, ISG15 and UBC8 are induced by interferon [36, 38], it would be therefore plausible that the correlation we observed was, in fact, due to enhanced interferon signalling. To determine this, we analysed if the correlation persisted with the upstream drivers, the type I interferons interferon alpha I and beta. Neither showed a correlation that was analogous to ISG15 and UBE2L6 (Fig. 1E, F). Other established interferon-induced genes such as IFITM1 and IRF3 (Fig. S1C, D), genes with an IFN-sensitive response element (ISRE) or Gamma interferon activation site (GAS), such as IFI16 or IFIT2 (Fig. S1E, F) failed to show the same correlation. These data suggest that the correlation between ISG15 and UBE2L6 and survival are not only due to augmented interferon signalling.

### ISGylation enhances cellular aggressiveness

**Fig. 2:**
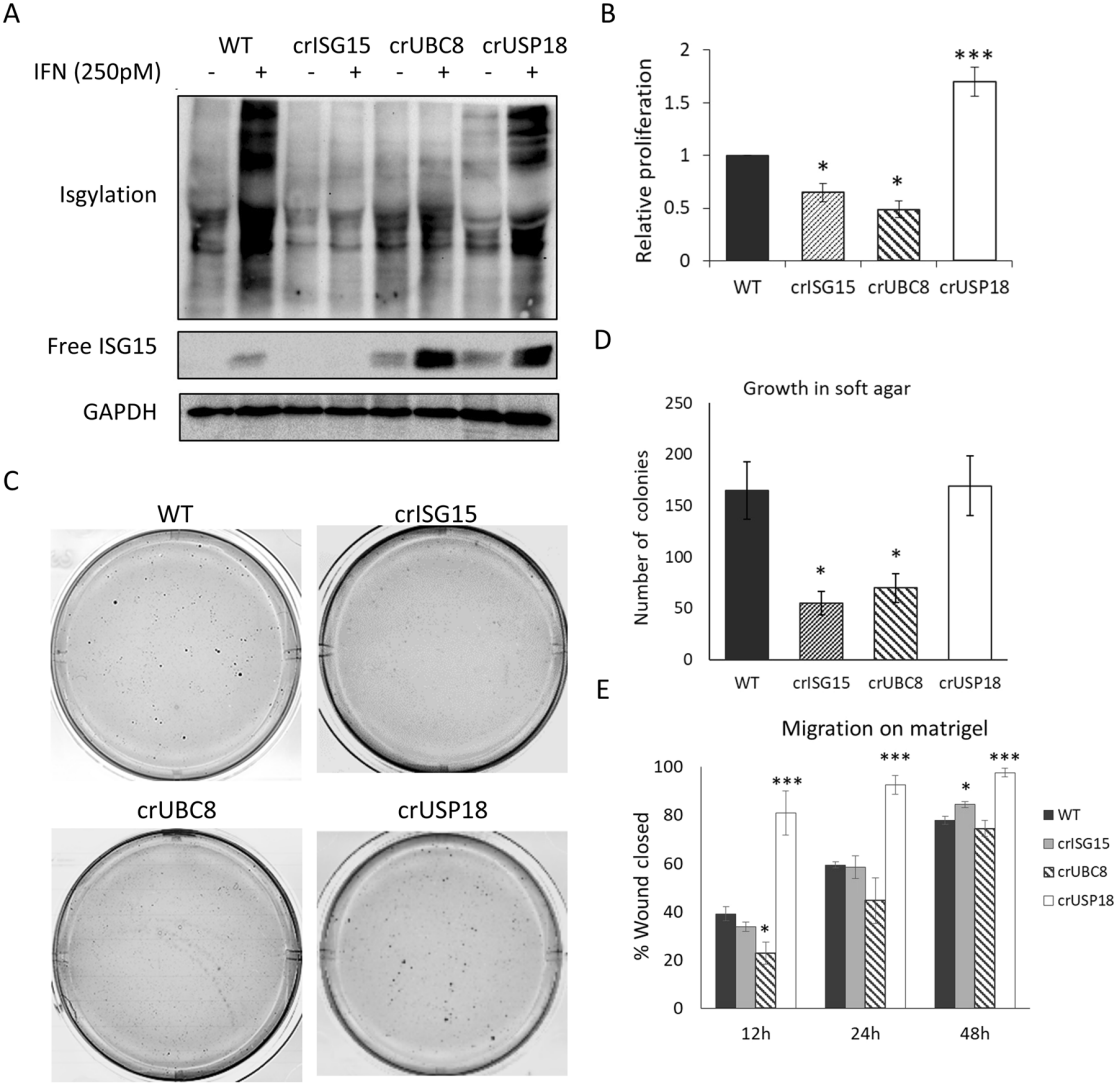
ISGylation correlates with an aggressive phenotype in MDA-MB-231-luc-D3H2LN. **A** WB analysis of free ISG15 and ISGylation levels in WT cells and the different representative clones, treated for 48 h with either vehicle or IFNb1a 250 pM. **B** Proliferation assay performed in the indicated clones. Equal amounts of cells were seeded and then counted manually. Bar graph shows the relative increase in cell number during 72 h ± S.E.M. *n* = 4. **C** Representative images of soft agar experiments. Equal cell numbers of the indicated, representative clones were seeded embedded in soft agar and after 28 days stained with crystal violet; *n* = 4. Images are displayed in grayscale for better identification of the colonies. **D** Quantification of number of colonies identified in the soft agar assay. Bar graph shows average ± S.E.M. **E** Relative migration. Equal amounts of cells were seeded into Matrigel-coated wells where scratch wounds were used to determine cell motility, photos were taken every 3 h for up to 48 h and motility was measured as percentage of wound closed per time point. Bar graph shows the average migration of the different clones versus WT cells at 12, 24 and 48 h, ±S.E.M; *n* = 3. *p* value < 0.05 (*), *p* value < 0.005 (***).

The inverse correlation of both ISG15 and UBC8 expression with disease-free survival suggested that conjugated ISG15 enhances metastasis and tumour progression. To study this, we generated different cell line models with varying levels of ISGylation in MDA-MB-231-luc-D3H2LN. Using CRISPR/Cas9 and two specific gRNAs per gene, we either knocked-out ISG15 (crISG15), UBE2L6 (crUBC8), USP18 (crUSP18) or generated a control line with the same Cas9 expression plasmid but without targeting gRNA (WT). These four cell lines allowed us to test the molecular and cellular characteristics of cells devoid of ISG15 (crISG15), devoid of ISGylation (crUBC8), with enhanced levels of ISGylation (crUSP18). Western blotting (WB) confirmed the respective knockouts (Fig. 1G). To increase basal levels of ISG15, USP18, UBC8 and ISGylation we additionally incubated the cells with IFN1b 250pM (or vehicle), which facilitated the confirmation of protein depletion (Figs. 1G, 2A). The result confirmed that crISG15 expressed no free or conjugated ISG15, crUBC8 expressed unconjugated ISG15 and, as additional bands are detected in the crUSP18 in non-interferon stimulated cells, that USP18 knock-out enhanced basal ISGylation levels. Surprisingly, the interferon-induced ISGylation profile of WT and crUSP18 cells was indistinguishable suggesting that the system is saturated under the experimental conditions. Due to the intricate relationship between IFNS and ISG15 or USP18 levels [39], all further experiments were performed in the absence of interferon allowing us to deconvolute both pathways. The analysis of MDA-MB-231 secretome [40, 41] failed to detect type-I interferons as secreted factors, suggesting that ISGylation is at basal levels and the effects observed in the cell lines were not due to differences in autocrine interferon secretion.

Having generated these models, we assessed if cellular phenotypes associated with tumour aggressiveness were linked to ISGylation. We focused on proliferation, anchorage-independent growth and cell motility or invasion. We found a positive correlation between ISGylation and the proliferation rate (Fig. 2B), the ability to form colonies in soft agar (Fig. 2C), the cell-density of those colonies (Fig. S2A), and their total number (Fig. 2D). Using the different clones and in the presence or absence of EGF or serum, we assessed the migration potential of the individual cell lines in a wound-healing assay. In this assay only crUSP18 cells, showed a statistically significant increase in motility at different time points (Fig. 2E), suggesting that enhanced ISGylation may increases motility when compared to basal levels. USP18 knock-out also increased the ability of cells to invade into Matrigel. In addition, we observed a trend indicating that cells devoid of ISGylation, crISG15 and crUBC8, had a reduced ability to invade (Fig. S2B, C). Overall, these data suggest that in vitro ISGylation increases several markers of tumour aggressiveness in a basal breast cancer cell line.

### ISGylation enables sustained Akt-signalling

**Fig. 3:**
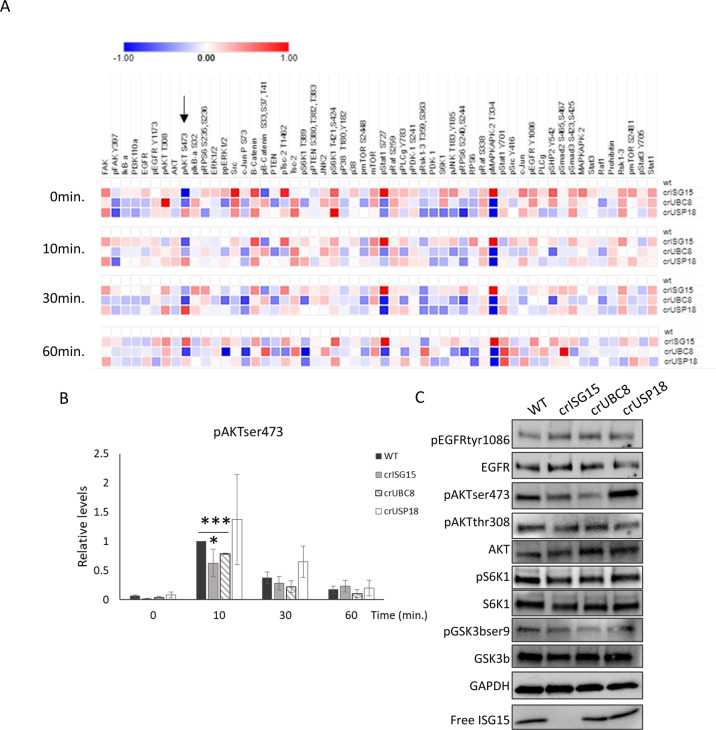
Loss of ISGylation is associated with decreased Akt phosphorylation. **A** RPPA analysis performed in WT and the different ISGylation CRISPR clones, treated with EGF 10 ng/ml for 0, 10, 30 and 60 min. Heatmap displays the Log2 of intensity normalised to WT at the different time points. Arrow indicates pAkt data. Averaged, *n* = 3 **B** Bar graph of pAkt intensities obtained in the RPPA. Values displayed are the mean ± S.E.M. *n* = 3. **C** Confirmation of RPPA data by WB analysis. Cells were treated with EGF 10 ng/ml for 10 min, WB and incubated with indicated antibodies.

ISG15 has been reported to regulate multiple signalling pathways, including Akt [42], ERK [27] or JAK/STAT [43]. To determine which are regulated by ISG15/UBC8-dependent-ISGylation, we employed a systematic approach. Using a Reverse Phase Protein Array (RPPA) we monitored how expression and phosphorylation of 58 signalling proteins were regulated at basal levels and upon EGF stimulation in the cell line panel (Fig. 3A). Despite not detecting significant changes in EGFR or EGFR phosphorylation levels between the clones, we identified downstream pathways that were regulated by ISGylation. As previously observed, cells lacking ISG15 had increased activated STAT1 [44]. In addition, we observed the increased expression of MAPKAPK2 [45]. Neither STAT1 nor MAPKAPK2 were altered in crUBC8 cells, it is therefore unlikely that UBC8-dependent ISGylation regulates these pathways. We further detected reduced ppERK levels in both crISG15 and crUBC8 at 10 min. when compared to WT and crUSP18, but the pathway most strikingly affected by ISGylation among all conditions was PI3K/Akt. pAktSer473 (pAkt) levels were positively correlated with ISGylation levels, with a maximal reduction of pAkt levels at 10 min. EGF stimulation in the crISG15 and crUBC8 compared to the control (Fig. 3B). In addition, we observed higher peak and sustained Akt phosphorylation in crUSP18 clones at 10 and 30 min. We confirmed the regulation of Akt and of the downstream pathway by WB (Figs. 3C and S3A). The decrease of pAkt was not due to decreased EGFR activation (Figs. 3C and S3A). We further validated the association between pAkt and ISGylation by re-/over-expression of ISG15 in crISG15 and WT cells which rescued or increased pAkt levels, whereas ISG15 overexpression did not alter pAkt in crUBC8 cells (Fig. S3B, C). These experiments demonstrated that sustained Akt signalling is enhanced by ISGylation rather than unconjugated ISG15.

To confirm that the suppression of pAkt upon ISGylation-loss was independent of EGFR, WT and crISG15 cells were treated with several concentrations of insulin, a strong activator the PI3K/Akt pathway. Like our observation with EGF, pAkt levels were suppressed in crISG15 compared to WT upon insulin stimulation, whereas receptor activation was unimpaired (Fig. S3D).

To determine if Akt suppression was due to altered feedback/network regulations, we performed time course experiments, stimulating the cells with EGF for 0, 2, 5, 10, 30 and 60 min. Our results (Fig. S3E, F) indicated that peak pAkt levels was unaffected. However, pAkt levels diverged depending on the ISGylation status, with pAkt suppressed at 10-, 30- and 60-minutes post-stimulation in clones lacking ISGylation. Conversely, Akt levels were augmented in crUSP18 cells at 10 min., suggesting that ISGylation influences the signalling sustenance rather than the acute activation.

### ISGylation is required for efficient receptor recycling

**Fig. 4:**
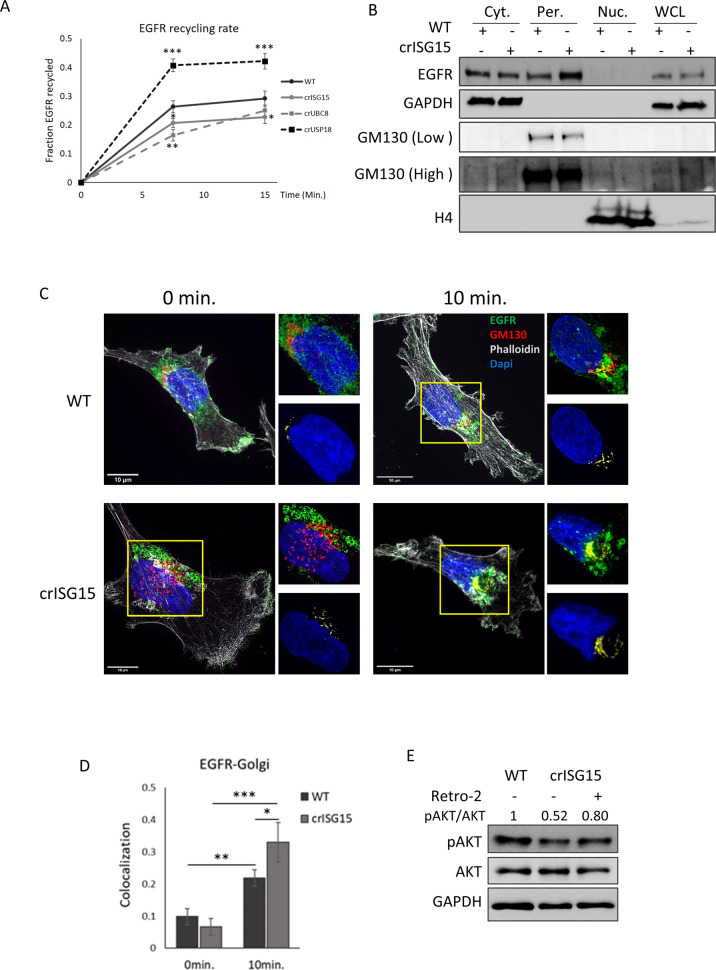
ISGylation promotes faster EGFR recycling. **A** Graph showing EGFR membrane recycling rate. Cells were surface labelled with NHS-SS-biotin, and surface receptors allowed to internalise for 30 min in serum free medium. Biotin remaining at the cell surface was removed, and internalised receptors allowed to recycle to the plasma membrane for the indicated time. Biotin label was removed from surface proteins at the cell membrane, cells were lysed and the fraction of recycled EGFR was determined at each timepoint. p-value < 0.05 (*), *p* value < 0.01 (**), *p* value < 0.005 (***). **B** Analysis by WB of EGFR localisation in WT and crISG15 cells stimulated with EGF for 10 min. Cells were fractionated into membrane and cytoplasm fraction (Cyt.), perinuclear region (Per.) and nucleus (Nuc.). EGFR levels detected by WB in the different fraction and the presence of the respective markers of subcellular localisation, GAPDH (cytoplasm), GM130 (Golgi) and Histone 4 (H4; Nucleus). Whole cell lysates (WCL) were included as reference of relative protein expression. **C** Representative super-resolution confocal images, obtained at 100 ×, of WT and crISG15 cells pre-treated with cycloheximide for 15 min. and stimulated for 10 min. with EGF 10 ng/ml. Cells were fixed, permeabilised and incubated with antibodies as indicated. Images show EGFR in green, GM130 as Golgi marker in red, phalloidin in grey and DAPI, in blue. At top right of each image, zoom of Golgi structure displayed. At bottom right of each image, visualisation of the co-localisation between EGFR and the Golgi marker GM130 of the images in yellow and DAPI, in blue as reference. 10 µm scale bars are displayed in the bottom-left corner. **D** Bar graph shows the average EGFR-GM130 co-localisation showed in **C** using Costes method, average ± SD; n = 8 field of view. *p* value < 0.05 (*) **E** Endosome to Golgi trafficking inhibition induces pAkt levels in crISG15 cells. WT and crISG15 cells treated for 10 min. with EGF. crISG15 cells were pre-treated with retro-2 for 15 min or not. Quantification of Akt activation, measured as pSer473-Akt/Akt signal, normalised to WT.

ISGylation has been shown to regulate PTEN stability [46], but we were unable to detect any regulation of PTEN protein expression (Fig. S3G), ruling this mechanism out. It has been previously shown that EGFR recycling is a crucial determinant of Akt signalling duration [47]. To explore if the reduction in sustained Akt activation was due to changes in trafficking, we assayed basal EGFR recycling using a surface biotinylation assay [48]. Reducing ISGylation decreased EGFR plasma membrane recycling, whereas EGFR recycled at a faster rate when ISGylation was enhanced by USP18 knockout (Fig. 4A). Loss of ISGylation could ‘trap’ EGFR in specific cellular compartments, which would in turn reduce recycling. To explore this, we used immunofluorescence to assay EGFR localisation in WT and crISG15 cells at basal and after 10 min. EGF stimulation (Fig.S4A). The latter timepoint was selected as it coincides with the most robust differences of Akt activation observed in the knock-out clones. In basal conditions there is a slight reduction of EGFR localising to the plasma membrane in crISG15 cells (Fig. S4B). However, the reduction of plasma membrane EGFR did not limit EGFR phosphorylation (Fig. 3A–C), suggesting that the concentration of EGF is rate limiting. However, after 10 min exposure, the differences in membrane-associated EGFR are more severe (Fig. S4B), which indicates that the defect in plasma membrane trafficking is exacerbated upon EGF stimulation. As expected, the EGFR localisation to early endosomes increased upon EGF stimulation, but this appeared to be independent of ISGylation (Fig. S4C). These data suggest that EGFR may be trapped in other compartments.

To assay ISGylation-dependent changes in EGFR subcellular distribution, WT and criSG15 cells were subjected to a fractionation protocol that generates a cytoplasmic fraction, containing the cytoplasm, cellular membrane and vesicles; a perinuclear fraction, containing the Golgi apparatus and endoplasmic reticulum, and a nuclear fraction. We detected that perinuclear EGFR was enhanced in cells lacking ISGylation (Fig. 4B).

The increased perinuclear localisation of EGFR in crISG15 cells could be due to increased shunting of EGFR towards the endoplasmic reticulum and/or the Golgi apparatus. To deconvolute de novo synthetised EGFR from EGFR expressed at the point of EGF stimulation, we pre-treated cells with cycloheximide, a protein translation inhibitor. Confocal imaging indicated that at 10 min. EGF stimulation in WT cells most of the internalised EGFR co-localised with EEA1-positive endosomal structures (Fig. S4A, C), however, a minority co-localised with GM130 (Fig. 4C), a Golgi apparatus marker. In crISG15 cells, we detected a statistically significant increase in the co-localisation between EGFR and GM130 (Fig. 4D). The increased localisation of EGFR in the Golgi in the absence of protein synthesis suggest that EGFR is trafficked and retained in the Golgi. Furthermore, we found that EEA1, EGFR and GM130 co-localised, indicative of increased retrograde transport (Fig. S4D). We also assessed if ISG15 affected EGFR shunting towards the lysosome by assaying co-localisation with CD63 and/or Lamp1 (Fig, S5A–C), but could not detect significant differences between WT and crISG15 cells.

To test if retrograde transport could impact Akt, we treated the cells with Retro-2, an inhibitor of the endosome-to-Golgi transport [49]. Retro-2 partially rescued pAkt and inhibited EGFR Golgi localisation in crISG15 cells (Figs. 4E, S6), suggesting that reducing EGFR trafficking to the Golgi salvages Akt activation in crISG15 cells.

These data show that ISGylation reduces the proportion of the receptor trafficked to the Golgi and promotes the return of the receptor to the plasma membrane.

### ISGylation does not have a direct effect on protein stability

To identify UBC8-dependent ISG15 substrate/s that control/s endosomal trafficking we devised two complimentary strategies. Firstly, ISG15 has been shown to regulate protein stability [28–30], thus ISG15 could alter expression levels of proteins controlling endosomal trafficking. Analysis of protein expression of the different knockout lines showed that 96 proteins were differentially expressed in the clones, when compared to WT cells (Table S1), three of which have been associated with protein trafficking (SEC61B, SEHIL, VAC14). However, none of these three proteins had expression level changes that were consistent with the phenotype observed in crISG15, crUBC8 and crUSP18, making them unlikely drivers. Having failed to identify a strong candidate, we devised a more targeted, secondary screen.

To map proteins covalently modified by ISG15, the ISGylome, we immunoprecipitated endogenous ISG15, determined which proteins co-immunoprecipitated with ISG15 and used crISG15 as negative control for ISG15 and crUSP8 as negative control for ISGylation. To prevent any non-covalent interactions, we lysed the cells under denaturing conditions prior to the immunoprecipitation step. Analysis of the data revealed 156 proteins as possible ISGylation targets, (Table S2). When compared with previous reported ISG15 interactors in the BIOGRID database, 92 were novel targets for ISGylation. Clustering of potential ISGylation targets using STRING (www.string-db.org) (Table S2) indicated that ISG15 is conjugated to proteins associated with a broad range of cell functions, including endosomal trafficking.

Surprisingly, when comparing the results from both screens, only one protein was in common, phosphoglycerate kinase 1. This suggests that: firstly, the effect ISGylation has on Akt signalling or endosomal trafficking is not related to protein stability, and secondly, that ISGylation does not primarily regulate protein stability under basal conditions.

### ISGylation reduces GDI2 affinity for Rabs

**Fig. 5:**
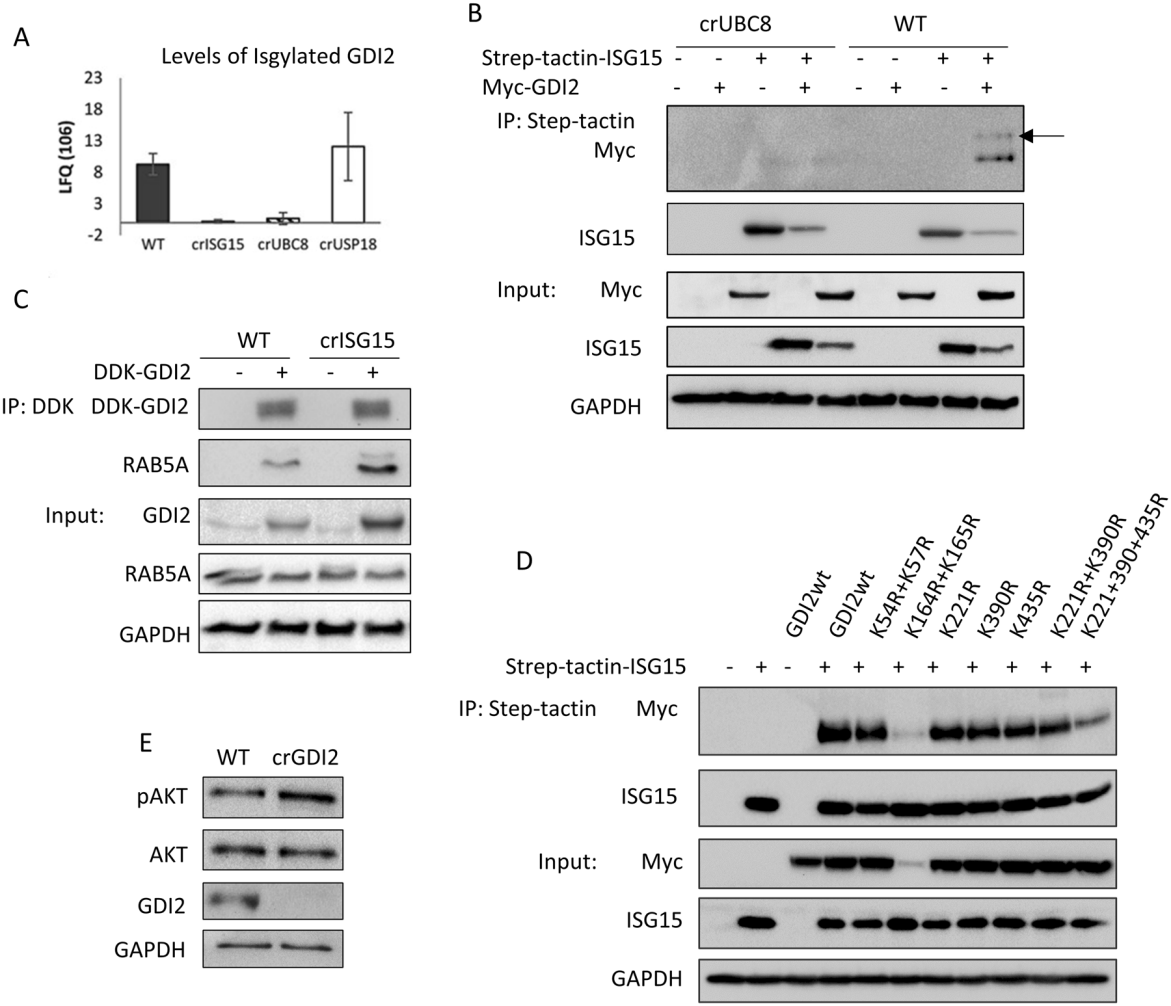
GDI2 is ISGylated and can regulate pAKT levels. **A** Label-free quantification (LFQ) values for GDI2 obtained from ISG15 pull-downs of the indicated clones, protein identification and quantification were performed using MaxQuant. **B** Analysis of GDI2 ISGylation using crUBC8 and WT cells transfected with empty vector, Step-tactin-ISG15, MYC-DDK-GDI2 or both, and subjected to a Step-tactin pulldown. WB show the pulldown and a 5% of the total lysates (Input). Arrow indicates ISGylated GDI2. **C** WB of WT and crISG15 cells transfected with MYC-DDK-GDI2, after 48 h cells were lysed and subjected to an anti-Flag pull-down. Blots show the pulldowns and a 5% of the total lysates (Input). **D** Analysis of putative GDI2 ISGylation sites. WT cells were transfected with Strep-tactin ISG15, MYC-DDK-GDI2, or Strep-tactin ISG15 with either MYC-DDK-GDI2wt or the indicated GDI2 mutants. After 48 h, cells were lysed and subjected to a Strep-tactin pull-down. The ISGylation status of the different GDI2 mutants was measured by determining the levels of GDI2 co-precipitated with ISG15. Blots show the results of the pulldown and a 5% of the total lysates (Input). **E** Analysis of Akt activation in crGDI2 by WB of WT and crGDI2 cells stimulated with EGF 10 ng/ml for 10 min. pAkt is pSer473.

One of the putative ISGylation substrates identified was GDP Dissociation Inhibitor 2 (GDI2) (Fig. 5A). GDI2 is a regulator of Rab activity and localisation making it a plausible integrator of ISGylation and endosomal trafficking. Additionally, GDI2 was described to be a putative target for ISGylation in two different studies [26, 50]. To confirm that GDI2 is ISGylated, we expressed Myc-DDK tagged GDI2, Strep-tactin tagged ISG15 or both, and determined the presence or absence of ISGylated GDI2. We detected the presence of exogenous GDI2 with a shift of the apparent molecular weight only in WT (Fig. 5B) confirming that GDI2 is ISGylated under basal conditions.

Neither the protein expression screen (Fig. S7A) nor a WB analysis of GDI2 levels (Fig. S7B) showed any changes in GDI2 protein levels in the different cell lines. Therefore, we hypothesised that ISGylation of GDI2 may be affecting GDI2 function rather than stability. To test this, we analysed the ability of GDI2 to interact with Rabs in the presence or absence of ISGylation. We transfected Myc-DDK-GDI2 into WT, crISG15, crUBC8, crUSP18 and analysed the interactome (Fig. S7C; Table S3). We found several Rabs to be interacting specifically with GDI2. Rab5 and Rab11, co-precipitated at higher levels with GDI2 in the cells where ISG15 or UBC8 had been knocked-out. In contrast, association of Rabs with GDI2 decreased further in crUSP18 cells. Surprisingly, transfected exogenous GDI2 was expressed at consistently increased levels in crISG15 cells (Table S3). However, this effect was absent in crUBC8, which indicates that this is not dependent on ISGylation. Rab5 and Rab11 are required for the maturation of early and recycling endosomes respectively [51, 52]. Rab5 is a key regulator of endosomal trafficking, making it a strong candidate to causally link the differences in EGFR recycling to ISGylation. To corroborate these results, we transfected WT and crISG15 cells with Myc-DDK-GDI2 and assayed endogenous Rab5 in the immunoprecipitate by WB (Fig. 5C). GDI2 co-precipitated higher levels of Rab5 in crISG15 cells when compared to WT, indicating that ISGylation inhibits the complex formation between GDI2 and Rab5, which in turn may affect EGFR recycling.

### ISG15 modified several lysine-residues of GDI2

To identify potential ISGylation sites on GDI2 we overexpressed GDI2 in COS1 cells and treated the cells with INFb1 to boost ISGylation. Analysis by LC-MS/MS indicated that lysine 435 (Fig. S7D, F) was modified by a double glycine peptide, a residual tag consistent with ISGylation. INFb1 treatment increased the level of the modification, suggesting that this may be an ISGylated residue. In addition, we mined databases for potential ISGylation sites. ISG15 and ubiquitin share the same Lysine–Glycine–Glycine (K-GG) peptide mark when digested with trypsin and are therefore undistinguishable from each other. We interrogated the Phosphosite database (www.phosphosite.org) for K-GG sites detected on GDI2 and found that peptides containing lysine 54, 57, 164, 165, 221 and 390 have been identified with the Ubiquitination/ISGylation marker.

To test if any of these sites could be ISGylated we substituted each lysine for arginine, creating the following mutants: K45R/K57R, K164R/K165R, K221R, K390R, K435R as well as combinations of several locations such as K221R/K390R and K221RK390R/K435R. Analysis by Strep-tactin-ISG15 pull-downs (Fig. 5D) showed that no single site mutant significantly decreases ISGylation. The double mutant, K164R/K165R, showed a marked decrease in ISGylation, but this was linked to a decrease in protein level, suggesting that the mutations were detrimental to protein expression/stability. A marked reduction in ISG15 conjugation was detected when GDI2 was mutated at K221, 390 and 435, suggesting that ISG15 can be conjugated to these sites.

**Fig. 6:**
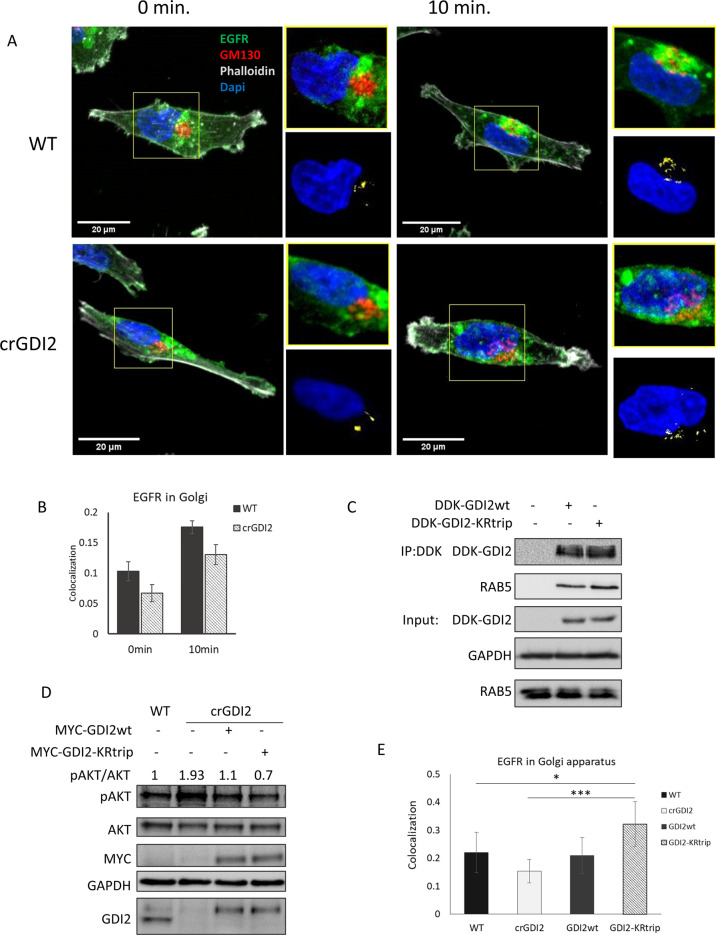
ISGylation of GDI2 reduces its activity and increases Akt activation. **A** Representative confocal images, obtained at 60x, of WT and crGDI2 cells pre-treated with cycloheximide for 15 min. and stimulated for 10 min. with EGF 10 ng/ml. EGFR in green, GM130 as Golgi marker in red, phalloidin in grey and DAPI, in blue. At top right of each image, zoom of Golgi structure displayed. At bottom right of each image, visualisation of the co-localisation between EGFR and the Golgi marker GM130 of the images in yellow and DAPI, in blue as reference. 20 µm scale bars are displayed in the bottom-left corner. **B** Bar graph shows the average EGFR-GM130 co-localisation showed in **A** using Costes method, average ± SD; *n* = 8 field of view. **C** WB of WT cells transfected with MYC-DDK-GDI2wt, or MYC-DDK-GDI2-KRtrip and after 48 h were lysed and subjected to Flag IP. GDI2 activity was measured by the levels of Rab5 detected in the pulldowns. Blots show the pulldowns and a 5% of the total lysates (Input). **D** Analysis of the effect GDI2-KRtrip has on Akt activation. WB of lysates from WT cells, crGDI2 cells, crGDI2 cells transfected GDI2wt or GDI2-KRtrip, treated for 10 min. with EGF. Values at in the upper part of the WB show the pAkt/Akt ratio as measure of Akt activation, normalised to the ratio in WT cells. **E** Quantification of EGFR-GM130 co-localisation displayed in Fig. S7. Bar graph of the average EGFR co-localisation with GM130 using Costes method ± SD of WT cells, crGDI2 cells, crGDI2 transfected with GDI2 expression vector, GDI2wt, or with the mutant GDI2, GDI2-KRtrip, treated for 10 min. with EGF. *n* = 8 fields of view. *p* value < 0.05 (*), *p* value < 0.005 (***).

We had observed that GDI2 binding to Rabs is enhanced in cells devoid of ISGylation and that absence of ISGylation impaired of Akt activation (Fig. 3A, B). To investigate if those two events are causally linked, we knocked-out GDI2 in MDA-MB-231-luc-D3H2LN cells (crGDI2). Firstly, we treated WT and crGDI2 cells with EGF. crGDI2 cells responded with a two-fold increased Akt phosphorylation compared to controls (Figs. 5E, S7E), suggesting that knockout of GDI2 enhances signalling through the PI3K/Akt pathway. We went on to determine how loss of GDI2 influenced EGFR trafficking to the Golgi by immunofluorescence (Fig. 6A) and observed a reduction of EGFR co-localising with the Golgi-marker GM130 (Fig. 6B). Secondly, our data suggested that ISGylation of GDI2 inhibits the ability of GDI2 to interact with Rabs. To test this, we transfected WT cells with either GDI2wt or the triple mutant K221RK390R/K435R (GDI2-KRtrip), immunoprecipitated GDI2, and assessed the ability to interact with Rab5. We detected a higher binding of Rab5 to GDI2-KRtrip (Fig. 6C), confirming that, despite mutating three K residues, the mutant retained the ability of GDI2 to bind to Rabs. Moreover, removing these ISGylation sites increased the ability of GDI2 to interact with Rab5. Thirdly, to tie these results together, we performed a rescue experiment by transfecting crGDI2 cells with either GDI2wt or GDI2-KRtrip to endogenous levels and analysed the phenotype. Re-expression of GDI2wt was able to reduce pAkt to a level similar to the WT cells (Fig. 6D). More interestingly, GDI2-KRtrip reduced pAkt levels further still.

To determine if the enhanced activation of Akt caused by GDI2 knockout is associated with reduced EGFR trafficking to the Golgi, we analysed EGFR localisation in WT cells, crGDI2 cells and in crGDI2 rescued by expressing GDI2wt or GDI2-KRtrip. We found that knocking out GDI2 reduced the presence of EGFR at the Golgi. This reduction was rescued by re-expressing GDI2. Intriguingly, rescuing crGDI2 with GDI2-KRtrip further increased the localisation of EGFR to the Golgi to a level beyond the WT cells (Figs. 5E and S8A, B).

Taken together, these experiments demonstrate that GDI2 is ISGylated, ISGylation of GDI2 inhibits binding to Rabs, and inhibiting GDI2 ISGylation suppresses Akt signalling by shunting EGFR towards the Golgi.

### ISG15 expression correlates with Akt-signalling in human tumours

**Fig. 7:**
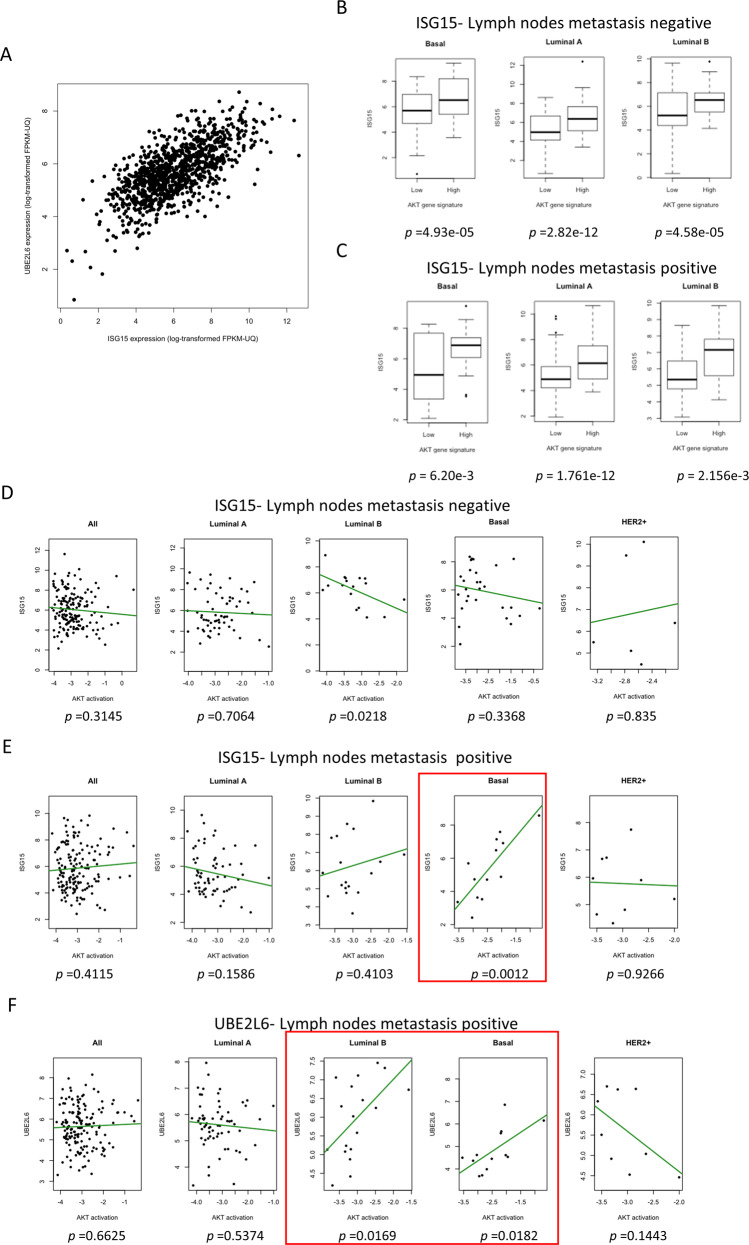
ISG15 correlates positively with Akt activation in breast cancers. **A** Correlation analysis of ISG15 and UBE2L6 mRNA expression levels in breast cancer samples. The spearman’s correlation is 0.66, *p* value < 2.2e-16 **B** Boxplots showing the correlation between an Akt-activity gene-signature and ISG15 mRNA in in lymph node negative basal breast cancer (*n* = 68), luminal A (*n* = 174), and luminal B (*n* = 41). *p* values are displayed below each graph. **C** Boxplots showing the correlation between an Akt-activity gene-signature and ISG15 mRNA in in lymph positive basal breast cancer (*n* = 36), luminal A (*n* = 188) and luminal B (*n* = 53). *p* values are displayed below each graph. **D** Correlation between pAkt and ISG15 mRNA levels in tumours without lymph node metastasis as a whole (*n* = 166) or classified by subtype; luminal A (*n* = 62), luminal B (*n* = 17), basal (*n* = 28) and HER2 (*n* = 6). **E** Correlation as in **D** in tumours positive for lymph node metastasis as a whole (*n* = 156) or classified by subtype: Luminal A (*n* = 65), Luminal B (*n* = 18), Basal (*n* = 13) and HER2 + (*n* = 9). **F** As **B** for UBE2L6 mRNA expression. In red boxes, tumour subtypes that showed a statistically significant correlation between pAkt and ISG15 or UBE2L6 mRNA.

To determine if ISG15/ISGylation and Akt signalling correlated in human breast tumours, we mined The Cancer Genome Atlas (TCGA) database (http://cancergenome.nih.gov/).TCGA has no protein expression data for ISG15 or UBC8, for this reason we used mRNA levels as surrogates. To establish if we could equate expression of ISG15 with ISGylation, we determined the correlation between ISG15 levels and UBC8 (Fig. 7A). Both genes were highly correlated, showing that high ISG15 could be taken as indicative of high ISGylation. With this assumption, we mined the database for a possible correlation between ISG15 mRNA levels and a PI3K/Akt activation gene signature [53]. The analysis revealed a significant correlation between ISG15 mRNA levels and Akt pathway activity in basal, luminal A and luminal B subtypes regardless of the lymph node status (Fig. 7B, C). In addition, TCGA includes a data set for which pAkt has been measured by RPPA. Here, ISG15 mRNA expression showed no correlation with pAkt in lymph node negative patients and a modest negative one for luminal B (Fig. 7D). However, in basal tumours with lymph node metastasis a positive correlation between ISG15 mRNA and pAkt was found (Fig. 7E) (*P* value = 0.0012). Interestingly, the same correlation was also found in for UBE2L6 mRNA in Luminal B (*P* value = 0.0169) and basal tumours (*P* value = 0.0182) with lymph node metastasis (Fig. 7F). This suggest that in human basal breast cancer UBC8-dependent ISGylation correlates with enhanced pAkt and signalling though the PI3K/Akt pathway.

Taken together, these data support the hypothesis that high levels of ISGylation mediated by UBC8 enhances the malignancy of breast cancer tumours by promoting Akt signalling. Mechanistically, we propose that ISGylation of GDI2 achieves this. ISGylated GDI2 has a lower affinity for Rab proteins, which in turn induces faster receptor recycling to the membrane by reducing the flux of EGFR towards the Golgi. Faster receptor recycling to the membrane then enables the tumour cells to sustain PI3K/Akt activation.

## Discussion

Metastasis is a main determinant of cancer mortality in solid tumours and an understanding of how the primary tumour acquires the ability to form secondary cancers is key to target the process. Altered EGFR-family RTK signalling is commonly associated with the induction of aggressive tumour characteristics, such as invasion, proliferation, or angiogenesis in breast tumours [54, 55]. To complement the picture, our data show that ISG15, UBE2L6 expression and prognosis are inversely associated in patients with lymph nodes metastasis. Interestingly, the lack of correlation of activators of the interferon-signalling pathway with survival in lymph node metastasis positive patients suggests that the correlation between UBC8-dependent ISGylation and survival is a distinct facet of global interferon signalling in tumours.

We demonstrate that ISGylation enhances EGFR recycling, effectively increasing membrane localisation and signalling. Several studies have also shown that increased localisation of EGFR in early endosomes increases the metastatic potential by enhancing signalling through the PI3K/Akt axis [48, 56, 57]. In this context it is unsurprising that expression of other regulator of EGFR endocytosis, such as Rab5, are markers of poor prognosis [58] and lymph node metastasis in breast cancers [59].

We show that ISGylation levels directly correlate with high recycling rates and decreased flux towards the Golgi apparatus. This effect is due to the ISGylation of GDI2, a regulator of Rab localisation and activity [60]. GDI2 contains two conserved domains, a protein-protein interaction domain, the Rab-binding platform, and a protein-lipid interaction domain, or lipid binding pocket, connected by the GDI effector loop [61]. We found that GDI2 ISGylation is not restricted to one residue. Of the three sites identified as ISGylated, lysine 221 may be the most relevant for the Rab interaction. Sequence alignments suggest that it is at the junction between the hinge and the lipid-binding domain, towards the side of the protein that interacts with Rabs. It is plausible that ISGylation of Lysine 221 could limit the interaction with the C-terminal prenyl group of the interacting Rab, the key step for the GDI-mediated extraction of Rab-GTPases from the membrane [62]. Further research is needed to establish how ISGylation of these residues regulates GDI2 activity. It is tempting to hypothesise that receptor trafficking is dependent on the balance between Rabs and GDIs, thus, in different cell types with different expression levels, ISGylation of GDI2 may affect RTKs mediated signalling distinctly. Furthermore, the analysis of the GDI2 interactome showed that the interaction of additional Rabs, was regulated by ISGylation. Consequently, absence of ISG15 could trigger other, unexplored defects, such as trafficking of *de novo* synthetised proteins from either the ER or Golgi, but further work is needed to test this.

## Materials and methods

Cell lines: MDA-MB-231 subclone D3H2LN, Cos1 and HEK293t were grown in DMEM 4.5 g/l glucose supplemented with 10% foetal bovine serum and 2 mM glutamine, at 37 °C and 5% CO_2_.

Proteomics: Changes in whole proteome levels were analysed using the Filter Aided Sample Preparation method, as previously described [63]. Interaction/ISGylation proteomics samples were analysed as described [64].

EGFR recycling: Recycling assays were performed as described previously [48].

## Supplementary information

Supplemental Text

Figure S1

Figure S2

Figure S3

Figure S4

Figure S5

Figure S6

Figure S7

Figure S8

Dataset Table S1

Dataset Table S2

Dataset Table S3
