# Supplementary material for: ISGylation drives basal breast tumour progression by promoting EGFR recycling and Akt signalling
Source: Oncogene. 2021 Sep 23;40(44):6235–47. doi: 10.1038/s41388-021-02017-8 (PMC8566238; doi:10.1038/s41388-021-02017-8)
Supplement: Supplementary file 1 — Supplemental Text [file 41388_2021_2017_MOESM1_ESM.docx]

**Supplementary Figure Legends**

**Fig. S1.** Kaplan-Meier plot of disease-free survival associated with mRNA levels of ISGylation associated machinery, in lymph node positive and negative patients for (A) UBA7 (n=744 and n=1183 respectively) and (B) UBE2E1 (n=744 and n=1183 respectively). Kaplan-Meier plot of disease-free survival associated with mRNA levels of interferon type I induced genes, in lymph node positive patients for (C) IFITM1 (n=724); (D) IRF3 (n=719); (E) IFI16 (n=619) and (F) IFIT2 (n=503).

**Fig. S2.** (A) Representative images of colonies obtained in soft-agar experiments at 20x. (B) Relative invasion. Equal amounts of cells were seeded, invasion ability was measured using a 3-D Matrigel scratch wound assay, photos were taken every 3h for up to 48h and invasion was determined as percentage of wound closure. Bar graph shows the average invasion of the different clones versus control cells at 12, 24 and 48h ±S.E.M; n=3. (C) Representative images of the invasion assay at 12, 24 and 48h.

**Fig. S3.** (A) Quantification of Fig. 3C. EGFR activation (pEGFRtyr1068) and AKT activation (pAKTser473). Values displayed are the mean ±S.E.M. (B) ISGylation profile of Fig. 3C. (C) WB of WT, crISG15 and crUBC8 cells non-transfected or transfected with a FLAG-ISG15 expression vector for 48h and treated with EGF 10 ng/ml for 10 min. Activation of Akt was detected by immunoblotting the levels of pAkt Ser473. (D) WB of WT and crISG15 cells treated with insulin, 0, 1, 10 or 100nM for 30 min. Activation of Akt and the Insulin pathway was detected by immunoblotting the levels of pAkt and pIRS-1. (E) WB analysis of Akt and ERK activation among the CRISPR clones treated with EGF 10 ng/ml for 0, 2, 5, 10, 30 or 60min. Activation of Akt was detected by immunoblotting the levels of pAkt; activation of ERK by immunoblotting the levels of ppERK. (F) Quantification of the time course for pAkt. Bars display the average value ±S.E.M of an n=4. (G) WB of the different clones blotted to analyse PTEN levels. p-value < 0.05 (*), p-value < 0.01, p-value < 0.005 (***).

**Fig S4.** (A) Representative super-resolution images, obtained at 100x, of WT and crISG15 cells stimulated for 0 or 10 min. with EGF 10 ng/ml. Cells were fixed, permeabilised and incubated with antibodies as indicated. Images show EGFR in green, early endosome antigen 1 (EEA1) in red, phalloidin in grey and DAPI, in blue. At top right of each image, EGFR channel is displayed. At bottom right of each image, visualisation of the co-localization between EGFR and the early endosome marker, EEA1, of the images in yellow and DAPI, in blue as reference. 10 µm scale bars are displayed in the bottom-left corner. (B) Bar graph shows the co-localization between EGFR and the cellular plasma membrane. Plasma membrane localization was determined using a mask against peripheral phalloidin staining. (C) Bar graph shows the average EGFR-EEA1 co-localization showed in (A) average ± SEM; n=12 field of view. . p-value < 0.05 (*); p-value < 0.005 (***). Co-localization expressed as Pearson correlation determined using Costes method. (D) Representative confocal images, obtained at 60x, of WT and crISG15 cells stimulated for 10 min. with EGF 10 ng/ml. Cells were fixed, permeabilised and incubated with antibodies as indicated. Images show EGFR in green, GM130 as Golgi marker in red, the early endosome marker EEA1 in grey and DAPI, used as a reference of the cell location, in blue. At top right of each image, zoom of Golgi structure displayed. At bottom right of each image, visualisation of the co-localization between EGFR and the Golgi marker GM130 of the images in yellow and DAPI, in blue as reference. 20 µm scale bars are displayed in the bottom-left corner. (E) Bar graph shows the average EGFR-GM130 co-localization showed in (D) using Costes method, average ± SD; n=8 fields of view. p-value < 0.05 (*).

**Fig. S5.** (A) Representative confocal images, obtained at 100x using super-resolution imaging, of WT and crISG15 cells stimulated for 0 or 10 min. with EGF 10 ng/ml. Cells were fixed, permeabilised and incubated with antibodies as indicated. Images show EGFR in green, GM130 in red, phalloidin in grey and DAPI, in blue. At top right of each image, zoom of the perinuclear structure is displayed. At bottom right of each image, visualisation of the co-localization between EGFR and GM130, depicted in yellow, and DAPI, in blue as reference. 10 µm scale bars are displayed in the bottom-left corner. (B) Bar graph shows the average EGFR-GM130 co-localization showed in (A) using Costes method, average ± SEM; n=9 fields of view. (C) Bar graph shows the average EGFR-Lamp1 co-localization in WT and crISG15 cells stimulated with EGF for 0 or 10min. using Costes method, average ± SEM; n=12 fields of view. (C) Bar graph shows the average EGFR-CD63 co-localization in WT and crISG15 cells stimulated with EGF for 0 or 10min. using Costes method, average ± SEM; n=12 fields of view.

**Fig. S6.** **Retro-2 inhibits EGF-induced Golgi localisation in crISG15 cells.** (A) Representative confocal images, obtained at 100x using super-resolution imaging, of WT and crISG15 cells stimulated for 10 min. with EGF 10 ng/ml in the presence of Retro-2 or DMSO. Cells were fixed, permeabilised and incubated with antibodies as indicated. Images show EGFR in green, GM130 in red, phalloidin in grey and DAPI, in blue. At the right a zoom of the perinuclear structure is displayed visualising of the co-localization between EGFR and GM130, depicted in yellow, and DAPI, in blue as reference. 10 µm scale bars are displayed in the bottom-left corner. (B) Bar graph shows the average EGFR-GM130 co-localization in WT and crISG15 cells stimulated with EGF for 10min in the presence of Retro-2 or DMSO. using Costes method, average ± SEM; n=12 fields of view.

**Fig. S7.** **ISGylation of GDI2 reduces its interaction with Rabs**. (A) Bar graph showing label-free quantification (LFQ) of GDI2 in whole-proteome analysis. Bars represent the average LFQ values ±SD. (B) WB of GDI2 the different clones (C) Bar graph showing the LFQ values of GDI2, ISG15, Rab5A and Rab11 obtained in MS analysis of GDI2 pull-downs in the indicated clones transfected with MYC-DDK-GDI2. Bars graphs display the average value ±SD. ISG15, Rab5A and Rab11 enrichment was determined by normalization of their LFQ values to GDI2. (D) Bar graph showing the relative quantification of K435 modified by GlyGly (E) Quantification of the pAkt levels in WT and crGDI2 cells treated with EGF for 10 min. Average values ±S.E.M; n=4, p-value < 0.01 (**). (F) Fragmentation spectra of the GDI2 K435 GlyGly peptide. Cos-1 cells were transfected with MYC-DDK-GDI2 or a control, treated with vehicle for 48h or transfected with MYC-DDK-GDI2 and treated with IFN1b 250Pm for 48h and then subjected to a FLAG immunoprecipitation, trypsin digestion and peptide purification. Peptides were analysed on a Fusion Lumos mass spectrometer. Identification of proteins and peptide modification was performed by the MaxQuant software.

**Fig. S8.** **GDI2 ISGylation regulates EGFR translocation to the Golgi apparatus.** (A) Representative confocal immunofluorescence images, at 60x, of WT cells, crGDI2, crGDI2 transfected with GDI2wt and crGDI2 cells transfected with GDI2-KRtrip and stimulated with EGF 10 ng/ml for 10 min. The channels show EGFR (green), the Golgi marker GM130 (red), early endosome marker EEA1 (grey), and nuclear marker, DAPI (blue). Zoom of Golgi structure displayed at the right of each image. 20 µm scale bars are displayed in the bottom-left corner. (B) Detailed view of EGFR and GM130 co-localization, visualisation of the co-localization between EGFR and the Golgi marker GM130 of the images displayed in (A) was performed by determination of co-localization areas using the Costes method. An EGFR-GM130 co-localization channel was built (yellow). DAPI (blue) is shown as a reference for the nucleus.

**Table S1. Differentially expressed proteins in the different ISGylation clones.** Datasheet shows the FASP data of hits with differential expression versus WT cells. Differences in expression were determined and ratio versus the WT and as statistical changes using LFQ values normalized by protease values.

**Table S2 Endogenous ISG15 pulldown MS analysis.** Datasheet 1 shows the specific hits for ISGylation. Determination of ISGylation was performed by enrichment of hits in samples from WT and crUSP18 cells versus the negative controls, crISG15 and crUBC8. Datasheet 2 shows the MaxQuant obtained data without further analysis to show ISG15 levels. Datasheet 3 shows KEGG pathways clusters using the STRING database (www.string-db.org/). p-values, FDR, number of hits and list of hits are displayed in the table.

**Table S3 Flag-GDI2 pulldown MS analysis.** Datasheet shows the specific hits for GDI2 interaction. Data was filter versus a negative control using the average LFQ, the obtained hits were normalized in each sample by their GDI2 LFQ values, and enrichment was performed to identify changes in interactome dependent of ISGylation status.

**Supplementary Materials and Methods**

Cell lines: MDA-MB-231 subclone D3H2LN, Cos1 and HEK293t were grown in DMEM 4.5 g/l glucose supplemented with 10% foetal bovine serum and 2mM glutamine, at 37ºC and 5% CO2.

Reagents: Recombinant Human Interferon Beta-1a was obtained from Prospec; EGF, insulin, ampicillin, kanamycin and puromycin were obtained from Sigma; transfection reagent TransIT-X2® was obtained from Mirus; transfection reagent jetPRIME was obtained from Polyplus transfections.

Plasmids: CAS9 expression plasmid (Cat. 49535), lentiviral envelope plasmid, pCMV-pVSV-G (Cat. 8454) and lentiviral packaging plasmid, psPAX2 (Cat. 12260), were obtained from Addgene; Human GDI2 expression vector pCMV6-DDK-Myc-GDI2, was obtained from Origene (Cat. RC200596.

Statistical methods and measurements: Unless otherwise is stated, two-tailed, unpaired t-test analysis was used to determine statistical significant changes. p-value < 0.05 (*), p-value < 0.01 (**), p-value < 0.005 (***). Unless otherwise stated bar graphs show the average (defined as the arithmetic mean) of the replicates (stated by n) of each experiment. Unless otherwise stated the error bars show the S.E.M. (Standard Error of the Mean) of the experiment replicates mean values.

Cloning and GDI2 mutagenesis: Knock-out cell lines were generated using CRISPR/CAS9. Two different guide sequences against the following genes were cloned into a lentiviral Cas9 expression vector, using the BSMB1 restriction sites: ISG15 (crISG15-1 Fw 5' GCTGGCGGGCAACGAATTCC 3' and crISG15-2 Fw 5' CTGCGTCAGCCGTACCTCGT 3'), UBE2L6 (crUBC8-1 Fw 5' CTGTCCGTTCTCGTCCACGT 3' and crUBC8-2 Fw 5' GGCTTGAACGGATACTCCGG 3') and USP18 (crUSP18-1 Fw 5' TCACGAATGAGCAAGGCGTT 3' and crUSP18-2 Fw 5' GCAAATCTGTCAGTCCATCC 3'), GDI2 (crGDI2-1 Fw 5' GCCACCCGAGTCAATGGGGA 3' and crGDI2-2 Fw 5' CACTCTCTCCTCCGTACGTA 3'). Guide sequences for ISG15, UBE2L6, and USP18 were obtained from [1]. Plasmids were transformed into Stbl3™ E. coli strain (Thermo Fisher).

Human ISG15 expressing plasmid was obtained by cloning ISG15 cDNA from MDA-MB-231 RNA, using the Forward primer 5’ATGGGCTGGGACCTGACGGTG 3’ and the reverse primer 5’ TTAGCTCCGCCCGCCAGGC 3’. PCR product was gel purified using the QuiaQUICK gel extraction kit and cloned into the pCR8/GW/ TOPOR entry vector. Plasmids were sequenced using the GW1 and GW2 primers included in the kit. ISG15 cDNA was subcloned into TAPE5-N destination vector 1 using LR Gateway Technology.

GDI2 mutagenesis was carried out using the Q5® Site-Directed Mutagenesis Kit (Promega, cat. E0554S) using manufacturer recommendations. The following primers for site-directed mutagenesis were designed using New England Biolabs online tool (<url://nebasechanger.neb.com/>): K54+54R ( Fw 5' TTTAGAATACCAGGATCACCACCC 3' and Re 5' TCTTCTGTATAAATCTTCCAATGGTG TTATAG 3'); K164+165R ( Fw 5' ATTGATCCTAGGAGGACCACAATGCGAGATGTGTATAAGAAATTTGAT 3' and Re 5' TGTGGTCCTCCTAGGATCAATGCCTTCAAAAGTTCTTGGATCTT 3'); for K221R ( Fw 5' AAGATATGGCAGAAGCCCATACC 3' and Re 5' GCCAAAGATTCACTGTAAAG 3'); K390R ( Fw 5' CCTGGTACCAAGAGACTTGGGAA 3' and Re 5' AGGTCACTGATGCTAACAAATTTC 3'); K435R ( Fw 5' TGAGGAAATGAGGCGCAAGAAGA 3' and Re 5' AAGTCAAACTCTGATCCTGTC 3').

Lentiviral production: Hek293t cells were transfected with 20µg of Cas9 expression vector, 2µg of the envelope plasmid pCMV-pVSV-G and 7.5µg of the packaging plasmid psPAX2, with TransIT-X2® (MirusBio) (2µl transfection reagent:1µg of plasmid). Media was harvested every 24h for 3 days, centrifuged (1500rpm, 3 min.), filtrated (0.45µm pore) and added to MDA-MB-231 D3H2LN cells. After 48h cells were selected to puromycin selection (2µg/ml), individual clones were grown and tested for protein expression. Two clones per guide were used in the experiments, treated as biological replicate.

Proliferation experiment: 2.5 10^4^ cells were seeded. Cell counting was performed each 12h for 3 days using a haemocytometer. Proliferation rate was determined as the slope of the variation in cell number (cell number increment) vs time. n=4.

Clonogenic assay: 6-well plates were coated with 1ml of 0.6% agar in DMEM and keep at room temperature. When solidified, 1 10^4^ cells in 1ml of DMEM, 0,3% agar, were applied to the top. Once solidified 1ml of media was applied on top. Media was changed twice per week, after 4 weeks colonies were detected using crystal violet staining. Plates images were taken and colony number quantify using Fiji using n=4 biological replicates. Migration and invasion assays: 5 10^4^ cells were plated per well in a IncuCyte® ImageLock 96-well (Essen Biosciences), 24h later a scratch-wound was made using the WoundMaker tool. For invasion assays, plates were matrigel-coated and after scratch wound was made, 100µl of matrigel (1mg/ml) was added on top of the cells. Phase-contrast images were taken every 3h, at 10x using a IncuCyte ZOOM system (Essen Biosciences). Invasion and motility were measured as percentage of wound closed per time point using IncuCyte ZOOM analysis software (n=3).

Immunoblotting: Cell were lysed in cold Lysis Buffer (0.1% triton x-100, 50 mM HEPES pH 7.5, 150 mM sodium chloride, 1.5 mM magnesium chloride, 1 mM EGTA, 1 mM sodium fluoride, 10mM Beta-glycerophosphate, 1 mM sodium vanadate, 10 µM leupeptin, 100 nM aprotinin, 1 mM PMSF).

Fractionation: Cells were lysed in cold hypotonic buffer (20mM Tris-HCl pH 7.5, 1mM MgCl_2_, 1mM EGTA, 10 mM sodium fluoride, 10 mM beta-glycerophosphate, 1mM sodium vanadate, 10 µM leupeptin,1mM PMSF) with NP-40 0.03% v/v (buffer A), for 3 minutes. Lysates were centrifugated at 800 G and the supernatant (cytoplasmic fraction) was transferred to a new tube. SDS and NP-40 (final concentration 0.1% and 1% v/v) were added to the cytoplasmic fraction. The pellet was washed once with buffer A with 0.03% NP-40 and once without detergent. Pellet was resuspended in cold lysis buffer B (100mM Tris-HCl pH 7.4, 1.5mM KCl, 2.5mM MgCl_2_, 0.2M LiCl, 10 mM sodium fluoride, 10 mM beta-glycerophosphate, 1mM sodium vanadate, 10 µM leupeptin,1mM PMSF ) containing sodium deoxycholate (0.1% v/v) and triton X-100 (0.1% v/v) and rotated at 4ºC for 15min. Lysates were centrifugated at 2000 g. Supernatant (perinuclear fraction) was transferred to a new tube and SDS, final concentration 0.1% v/v, was added to the perinuclear fraction. Pellets were washed thrice with buffer B with detergents. The pellet was resuspended in RIPA buffer and sonicated. After centrifugation at 17000xg 4ºC for 15min. the supernatant (nuclear fraction) was transferred to a new tube.

Immunoblot: Cleared lysates were resolved on SDS-PAGE acrylamide gels and transferred onto PVDF membranes (Whatman) using Mini Trans-Blot® Electrophoretic Transfer Cell (BIO RAD). Membranes were blocked in 4% BSA for 1h and blotted with primary antibodies (see paragraph below). Immunocomplexes were visualized using ClarityTM Western ECL Substrate (BioRAD) in a ChemidocTM MP (BioRAD) with horseradish peroxidase–conjugated secondary anti-bodies (CST 1:10000). WB quantification was performed using ImageJ. All immunoblots experiments were repeated at least three times (n=3).

Primary antibodies: Anti-ISG15 antibody (1:1000) was obtained from PBL Assay Science; antibodies against GDI2 (1:2000) were obtained from Proteintech and Life Technologies; antibody against USP18 (1:500) was obtained from Proteintech; antibodies anti pEGFR Tyr1086 (1:2000), pAkt Ser473 (D9E) (1:2000), Akt (1:1000), pGSK3b Ser9 (S9) (1:2000), GSK3b (27C10) (1:2000), pS6K1 Thr421/Ser424 (1:1000) , S6K1 (49D7) (1:1000), MYC-tag (71D10) (1:1000), Rab5 (C8B1) (1:1000), PTEN (D4.3) (1:3000) and GAPDH (14C10) (1:3000) were obtained from CST; antibody against pAkt Thr308 (1:500) was obtained from Millipore; antibody against IRS1 (1:500) was obtained from Abcam; antibody against pIRS1 (Tyr612) (1:250) was obtained from Invitrogen; antibodies against HRP-FLAG® (1:3000), ppERK Thr202/Thr185,Tyr204/Tyr187 (1:3000) and ERK (1:3000) were obtained from Sigma; antibodies against UBC8 (K1H3) (1:500) and EGFR (C-30) (1:3000) were obtained from Santa-Cruz.

Reverse phase protein array (RPPA): Biological triplicates (n=3) of each construction, for each time point, were lysed with RPPA lysis buffer (1% triton x-100, 50 mM HEPES (pH 7.5), 150 mM sodium chloride, 1.5 mM magnesium chloride, 1 mM EGTA, 10 mM sodium fluoride, 10 mM beta-glycerophosphate, 1 mM sodium vanadate, 10% glycerol, 10 µM leupeptin, 100 nM aprotinin). Cleared lysates were normalized to 1.5mg/ml and four serial dilutions of each sample, were spotted onto nitrocellulose-coated slides (Grace Bio-Labs) in technical duplicates. Primary antibodies were applied at 1:250 concentration. Bound antibodies were detected by incubation with DyLight 800-conjugated secondary antibody (New England BioLabs). An InnoScan 710-IR scanner (Innopsys) was used to read the slides, and images were acquired at the highest gain without saturation of the fluorescence signal. The relative fluorescence intensity of each sample spot was quantified using Mapix software (Innopsys). The signal intensity of each antibody was determined using the linear regression made with the median intensity of the four different sample dilutions. Signal intensity were normalised to total protein loaded, determined by staining a slide with fast-green, a non-specific protein dye and scanning at 790Um.

Whole-proteome analysis: Changes in whole proteome levels were analysed using the Filter Aided Sample Preparation (FASP) method, as previously described [2]. Briefly, biological triplicates (n=3) were lysed in FASP Lysis buffer (50 mM HEPES (pH 7.5), 150 mM sodium chloride, 1% Triton X-100,1% SDS, 1.5 mM magnesium chloride, 1 mM EGTA, 10 mM sodium fluoride, 10 mM beta-glycerophosphate, 1 mM sodium vanadate, 10 µM leupeptin, 1mM PMSF), sonicated and centrifuged at 15000rpm 15min at 4°C. 100 µg of proteins were, reduced, alkylated and subjected to Lys-C (1:100 w/w) and trypsin (1:100 w/w) digestion. Peptide concentration were estimated by measuring 280 nm absorption using a Nanodrop ND-1000.

10 µg of peptides were purified using the stage-tip protocol [3]. The eluted peptides were lyophilized in a Concentrator Plus (Eppendorf), resuspended in 0.1% TFA and analysed by LC-MS/MS on a Q Exactive Mass Spectrometer (Thermo Fisher). Protein identification and quantification was performed by label-free quantification using the MaxQuant software suite with, carbamidomethyl cysteine set as a fixed modification and methionine oxidation and protein N-terminal acetylation as variable modification [4]. LFQ were normalized and statistical changes were determined using an adjusted t-test against control cells with a p-value 0.05.

Pulldowns: For ISGylome analysis cells were lysed in Lysis Buffer (see Immunoblotting section) with 1% Triton X-100 and 1% SDS and the cleared lysate were 10-fold diluted with detergent-free Lysis Buffer. Pulldowns were performed in a KingFisher Duo (Thermo Scientific). Cleared lysates were incubated with 5µL of protein G Mag Sepharose™ Xtra beads (GE Healthcare) and 1µg of antibody anti-ISG15 per sample for 4h, washed two times Lysis Buffer and three times in TBS and resuspended in 100 µl digestion buffer (2 M urea, 50 mM Tris-HCL pH7.5, 1 mM DTT) with 0.25µg of porcine trypsin MS-grade (Promega). Samples were digested at 37ºC for 8 hours. Cysteines were alkylated with iodacetamide (1mg/ml), 30min. and acidified with TFA (final concentration 1% v/v). Peptides were desalted and purified using C18 STAGE tips [3] . The eluted peptides were lyophilized in a Concentrator Plus (Eppendorf), resuspended in 0.1% TFA and analysed by LC-MS/MS on a QExactive+ Mass Spectrometer (Thermo Fisher). Label-free quantification approach was used for protein identification and quantification using MaxQuant software [4].

GDI2- ISGylation analysis using exogenous ISG15 was performed by incubating cleared lysates with 8µl strep-tactin MagStrep "type3" XT beads (IBA Biosciences) for 2h, washed two times in lysis buffer and one time in TBS and resuspended in SDS loading buffer without reducing agents.

GDI2 Pulldowns: 10cm dishes Cell were lysed in Lysis buffer (see Immunoblotting section above); cleared lysates were incubated with 5µl Anti-FLAG® M2 Magnetic Beads (SIGMA) for 2h and washed two times in Lysis Buffer and one time in TBS. For Immunoblot analysis, beads were resuspended in loading buffer supplemented with 1 mM DTT. For MS analysis, beads were additionally washed two times with TBS and resuspended in digestion buffer (2 M urea, 50 mM Tris-HCl pH7.5, 1 mM DTT) with 0.25µg of porcine trypsin MS-grade (Promega) and processed as above. The eluted peptides were lyophilized in a Concentrator Plus (Eppendorf), resuspended in 0.1% TFA and analysed by LC-MS/MS on a QExactive or Fusion Lumos (Thermo Fisher) as described [3]. Label-free quantification approach was used for protein identification and quantification using MaxQuant software [4].

For MS-pulldown experiments, LFQ values were normalized to the bait (either ISG15 or GDI2) intensities and fold enrichment were calculated as the ratios of the averaged, normalised LFQs across two biological replicates.

Imaging: Cells were seeded onto a high-performance glass coverslip (Zeiss), starved overnight and treated for 10min. with EGF (10ng/ml) or kept without EGF stimulation. For EGFR-Golgi co-localization studies cells were pre-treated with cycloheximide (CHX) for 15min. before EGF stimulation. Cells were washed twice with cold PBS, fixed and permeabilised with Fixation buffer (3.7% formaldehyde, 0.1% NP-40 in 50mM Pipes pH6.8, 125mM NaCl, 10mM MgCl2, 1mM EGTA) for 5min. and blocked in TBS, 2% BSA for 1h. Coverslips were incubated overnight with primary antibodies (see paragraph below). Slides were washed twice with TBS and then incubated for 1 h at room temperature with secondary antibodies anti-mouse Alexa-546 or anti-rabbit-488, (Life Technologies) at 1:250 for confocal microscopy. For super resolution microscopy secondaries F(ab´)2 anti-rabbit alexa 594, 488 and anti-mouse alexa 647 at 1:400 were used (Life Technologies). Slides were washed twice with TBS and incubated with DAPI (1:100) or DAPI and rhodamine-phalloidin (1:200), for 5 minutes, washed two times with TBS and mounted using VECTASHIELD antifade mounting media (Vector labs) for confocal or Abberior liquid antifade (Abberior) for super-resolution microscopy. Stacks for confocal imaging were taken with an Olympus FV1000 and FV3000 confocal, with 60x oil objective. For super-resolution imaging, image stacks were taken with a NIKON CSU-W1, SORA, with a 100x oil objective. Co-localization analysis were performed slice by slice using Imaris 7.7 software, images were subjected to denoise and background subtraction. Co-localization was determined as the Pearson correlation between channels using the Costes method [5] included in the ImarisColoc package. For cellular membrane, early endosome and Golgi apparatus, object mask were generated to determine the objects volume. For confocal experiments eight fields of view per condition (n=3) were used, only whole imaged-cells were used for subsequent image analysis. For super resolution images twelve fields of view were used.

Primary antibodies: rabbit anti EGFR-Alexa488 (1:100),mouse anti GM130 (1:200) and rabbit anti Lamp1-Cy3 were obtained from Abcam; rabbit anti GM130 -Alexa647 (1:100) was obtained from Thermo Scientific); mouse anti EEA1 (1:200) was obtained from BD Biosciences; rabbit anti EGFR (1:100) was obtained from CST; Mouse anti CD63 was obtained from Novus Bio.

EGFR recycling: Recycling assays were performed as described previously (Caswell et al., 2008). Briefly, cells were serum starved for 30-45 minutes prior to surface labelling labelled at 4°C with 0.13mg/ml NHS-SS-Biotin (Pierce), and biotinylated receptors internalized for 30 min. at 37°C in serum free medium before removal of remaining surface biotin by reduction with sodium 2-mercaptoethane sulfonate at 4°C. Internalised receptors were allowed to recycle to the cell surface by returning the cells to 37°C. Surface biotin removed a second time by a reduction step at 4°C and the fraction of biotinylated, internalised receptors was analysed by ELISA. The antibodies used for capture ELISA detection of EGFR were obtained from BD Biosciences. n=4.

Statistical analysis of Akt signalling and ISG15 expression in human breast tumours: Data acquisition and pre-processing: Publicly available RNA-seq data, in FPKM format, from breast tumours in the TCGA-BRCA project, was downloaded from the genomic data commons (https://portal.gdc.cancer.gov/). Non-expressing genes were excluded and FPKM counts were upper quartile normalised, scaled and log2(x+1) transformed. Breast cancer subtypes were defined using oestrogen receptor, progesterone receptor and HER2 status reported by TCGA. All analyses were restricted to female, primary breast cancer (N=1078). Lymph node involvement status was also obtained for each patient (N positive = 463, N negative = 450).

Akt gene signature: We built a gene signature for Akt activation based on genes identified by Creighton *et al.* [6]. Principal component analysis was conducted on the expression of these genes to identify the pattern of gene expression that explained the greatest variance in the data. The first principal component was used as the Akt gene signature that was then split into high and low groups at the median for visualisation. The correlation between ISG15 expression and the Akt signature was assessed using linear regression for each breast cancer subtype with and without lymph node involvement separately.

Akt activation from RPPA: pAkt measured by RPPA was available for a subset of the patients included above (N=322, N node positive = 156, N node negative = 166). The relationship between ISG15 and UBE2L6 expression and Akt activation was examined using linear regression for each subtype and in node positive and node negative cancer separately. The Benjamini-Hochberg adjustment was used to interpret the results while accounting for the effect of multiple testing. All analyses were carried out in R (version 3.6.0).

1 Shalem O, Sanjana NE, Hartenian E, Shi X, Scott DA, Mikkelson T *et al*. Genome-scale CRISPR-Cas9 knockout screening in human cells. *Science* 2014; 343: 84-87.

2 Wisniewski JR, Zougman A, Nagaraj N, Mann M. Universal sample preparation method for proteome analysis. *Nat Methods* 2009; 6: 359-362.

3 Turriziani B, Garcia-Munoz A, Pilkington R, Raso C, Kolch W, von Kriegsheim A. On-beads digestion in conjunction with data-dependent mass spectrometry: a shortcut to quantitative and dynamic interaction proteomics. *Biology (Basel)* 2014; 3: 320-332.

4 Cox J, Mann M. MaxQuant enables high peptide identification rates, individualized p.p.b.-range mass accuracies and proteome-wide protein quantification. *Nat Biotechnol* 2008; 26: 1367-1372.

5 Costes SV, Daelemans D, Cho EH, Dobbin Z, Pavlakis G, Lockett S. Automatic and quantitative measurement of protein-protein colocalization in live cells. *Biophys J* 2004; 86: 3993-4003.

6 Creighton CJ. A gene transcription signature of the Akt/mTOR pathway in clinical breast tumors. *Oncogene* 2007; 26: 4648-4655.
