# Supplementary figures and images for: ISGylation drives basal breast tumour progression by promoting EGFR recycling and Akt signalling
Source: Oncogene. 2021 Sep 23;40(44):6235–47. doi: 10.1038/s41388-021-02017-8 (PMC8566238; doi:10.1038/s41388-021-02017-8)

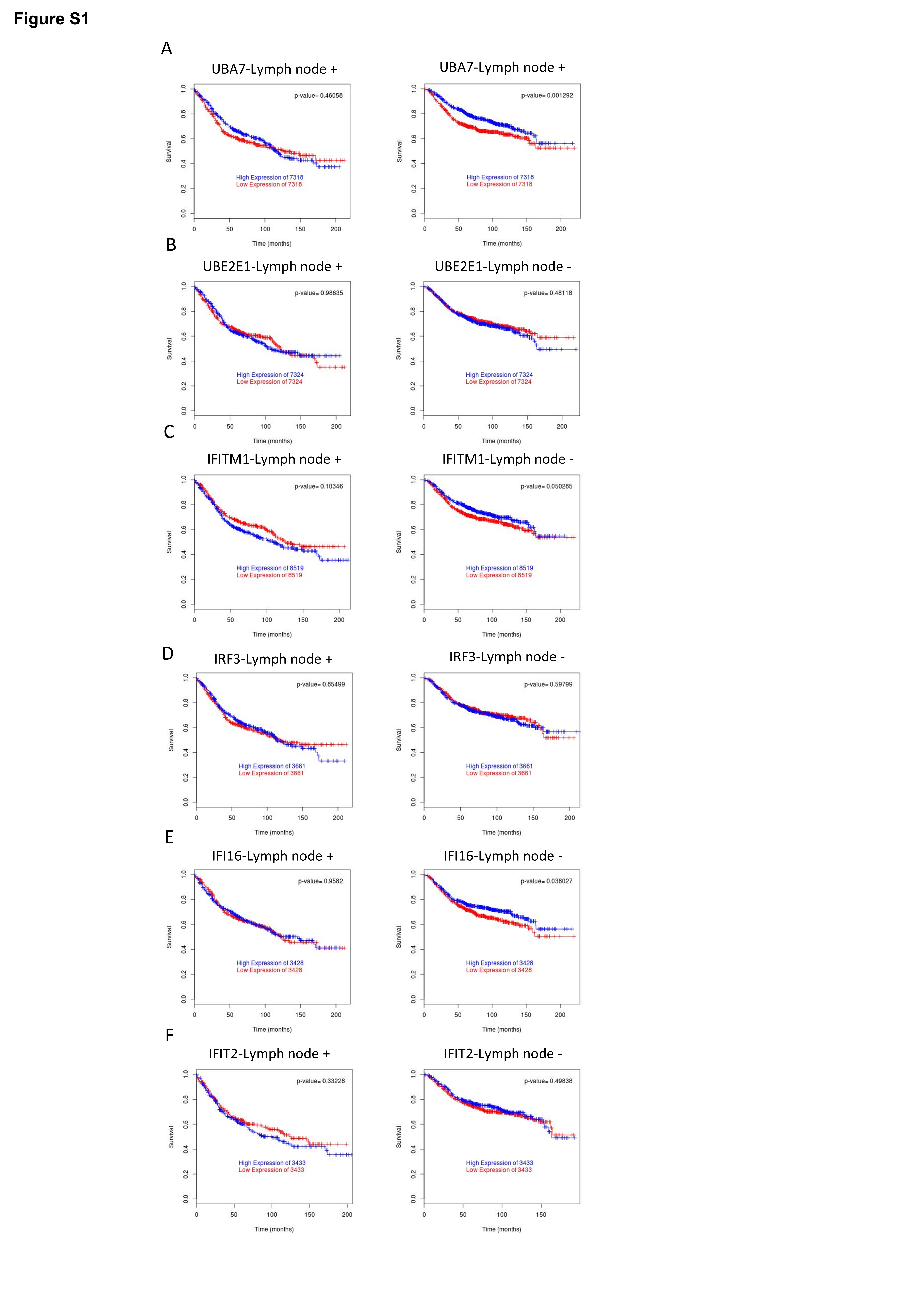

Supplement: Supplementary file 2 — Figure S1 [file 41388_2021_2017_MOESM2_ESM.tif]

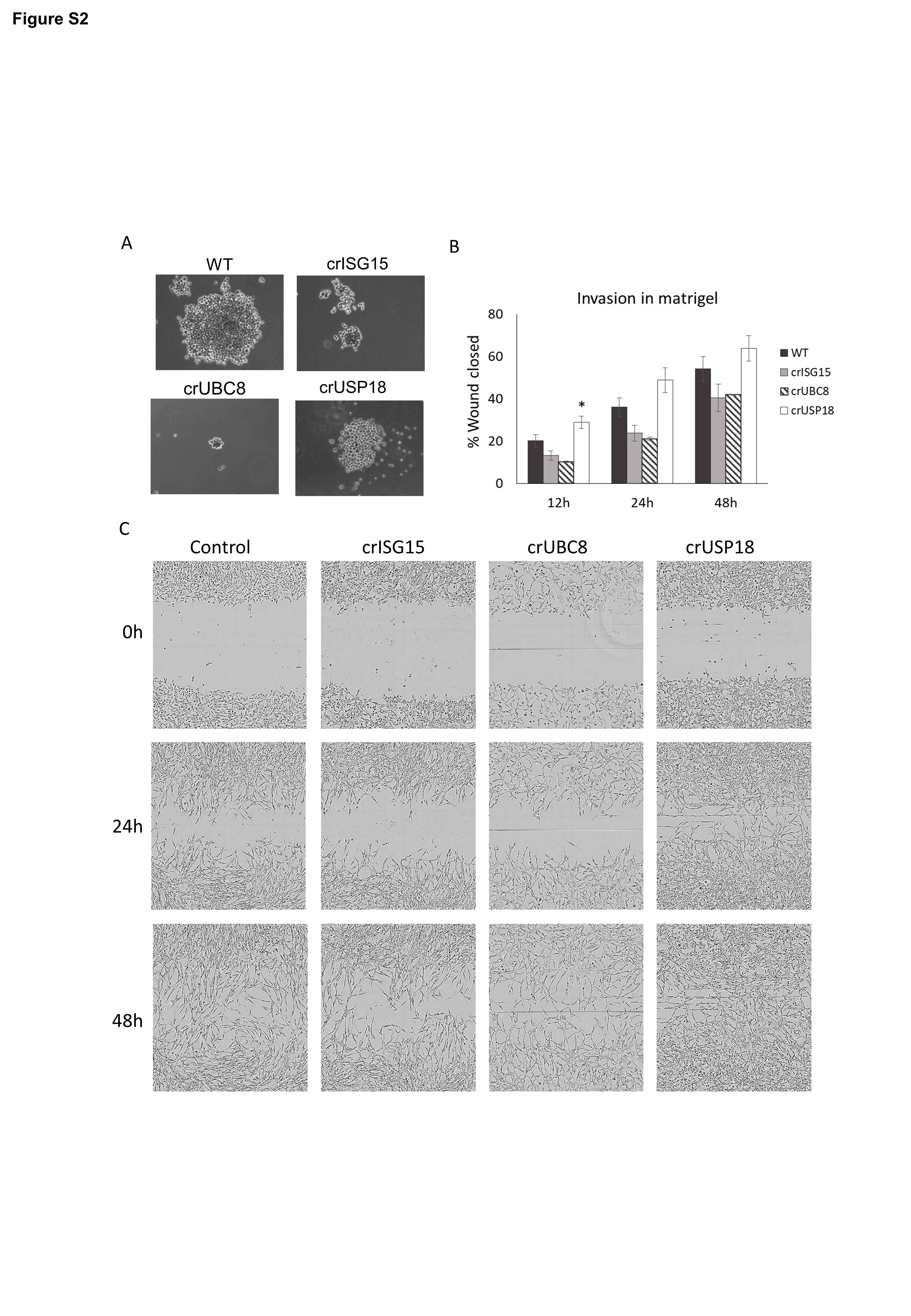

Supplement: Supplementary file 3 — Figure S2 [file 41388_2021_2017_MOESM3_ESM.tif]

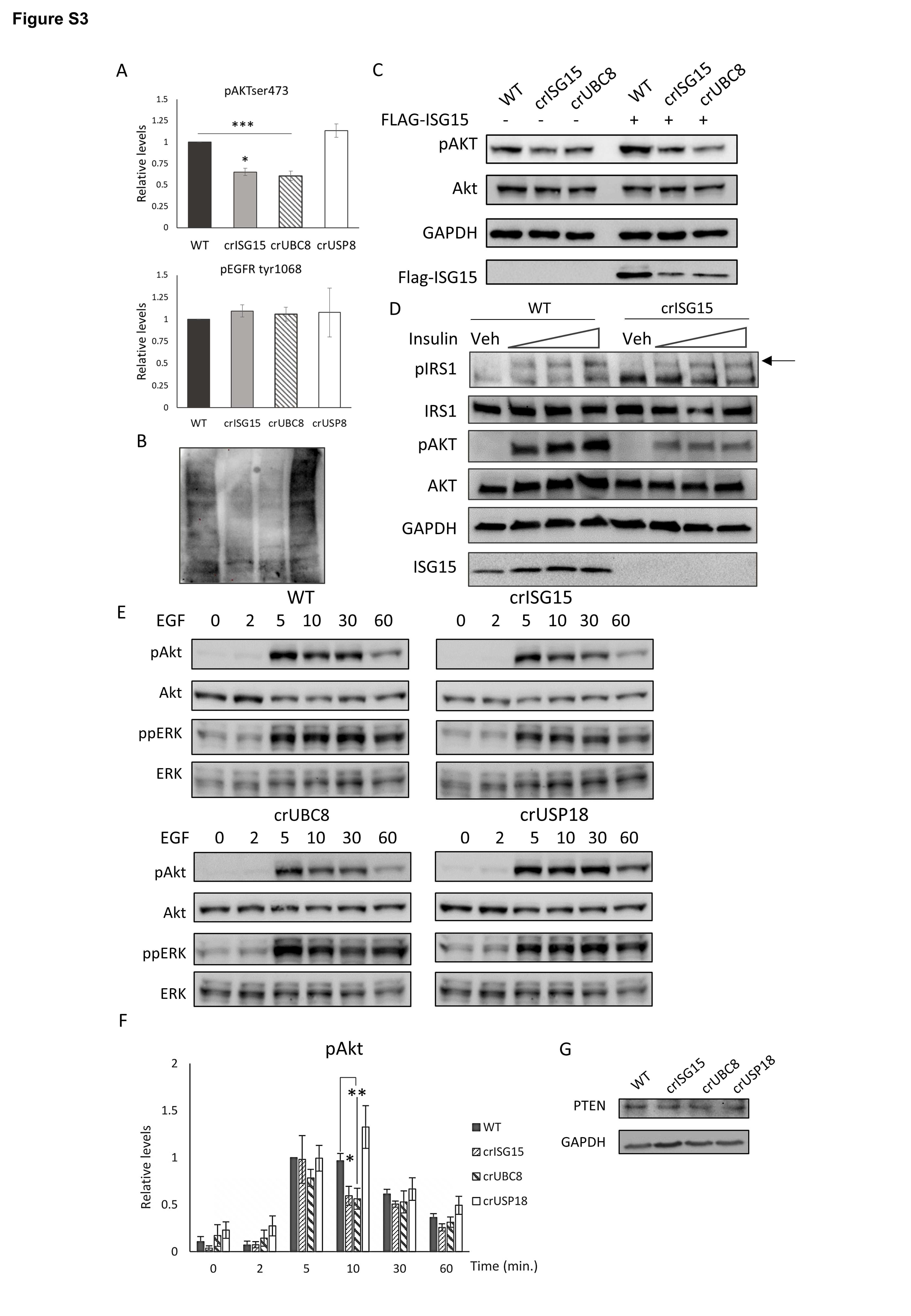

Supplement: Supplementary file 4 — Figure S3 [file 41388_2021_2017_MOESM4_ESM.tif]

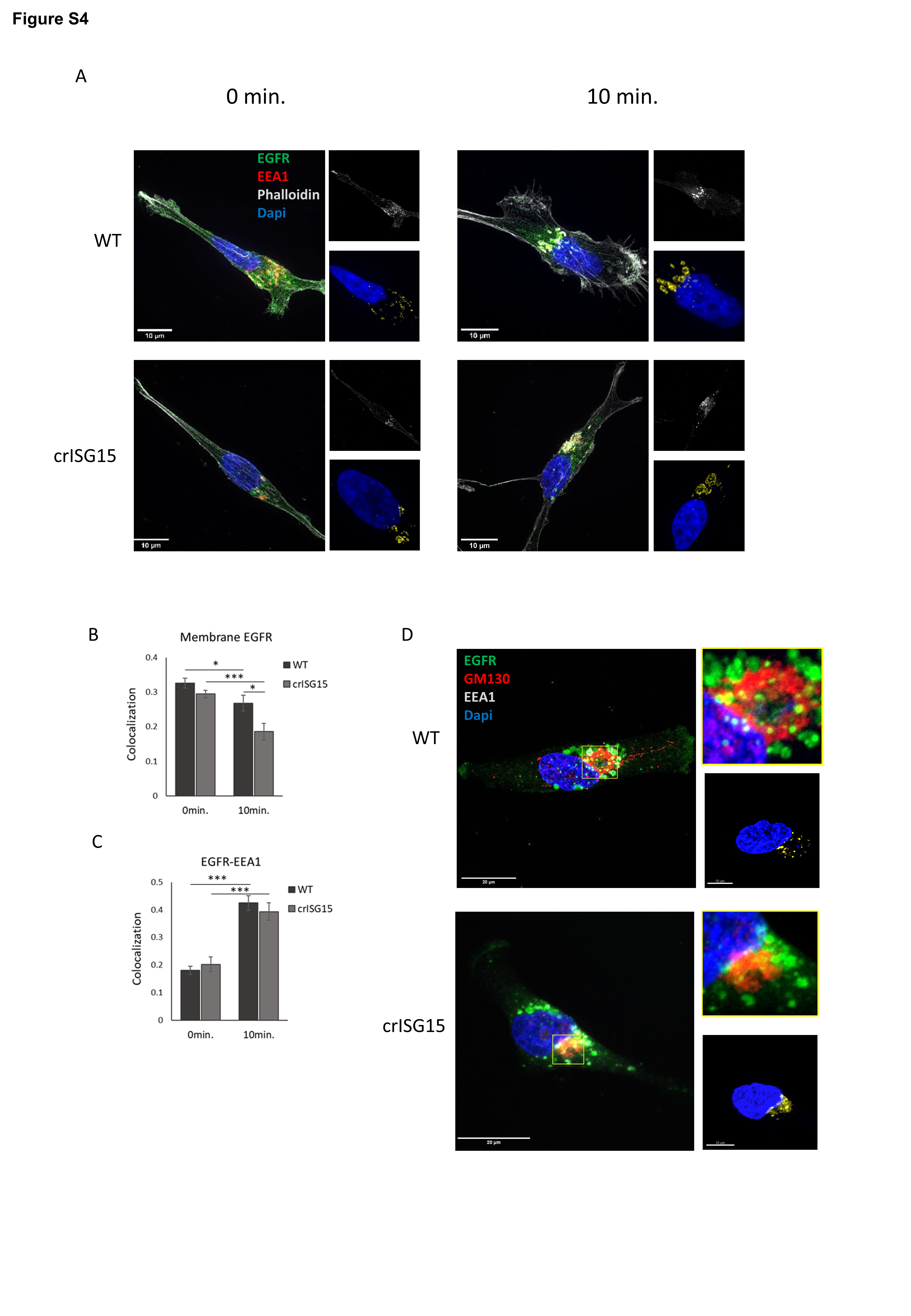

Supplement: Supplementary file 5 — Figure S4 [file 41388_2021_2017_MOESM5_ESM.tif]

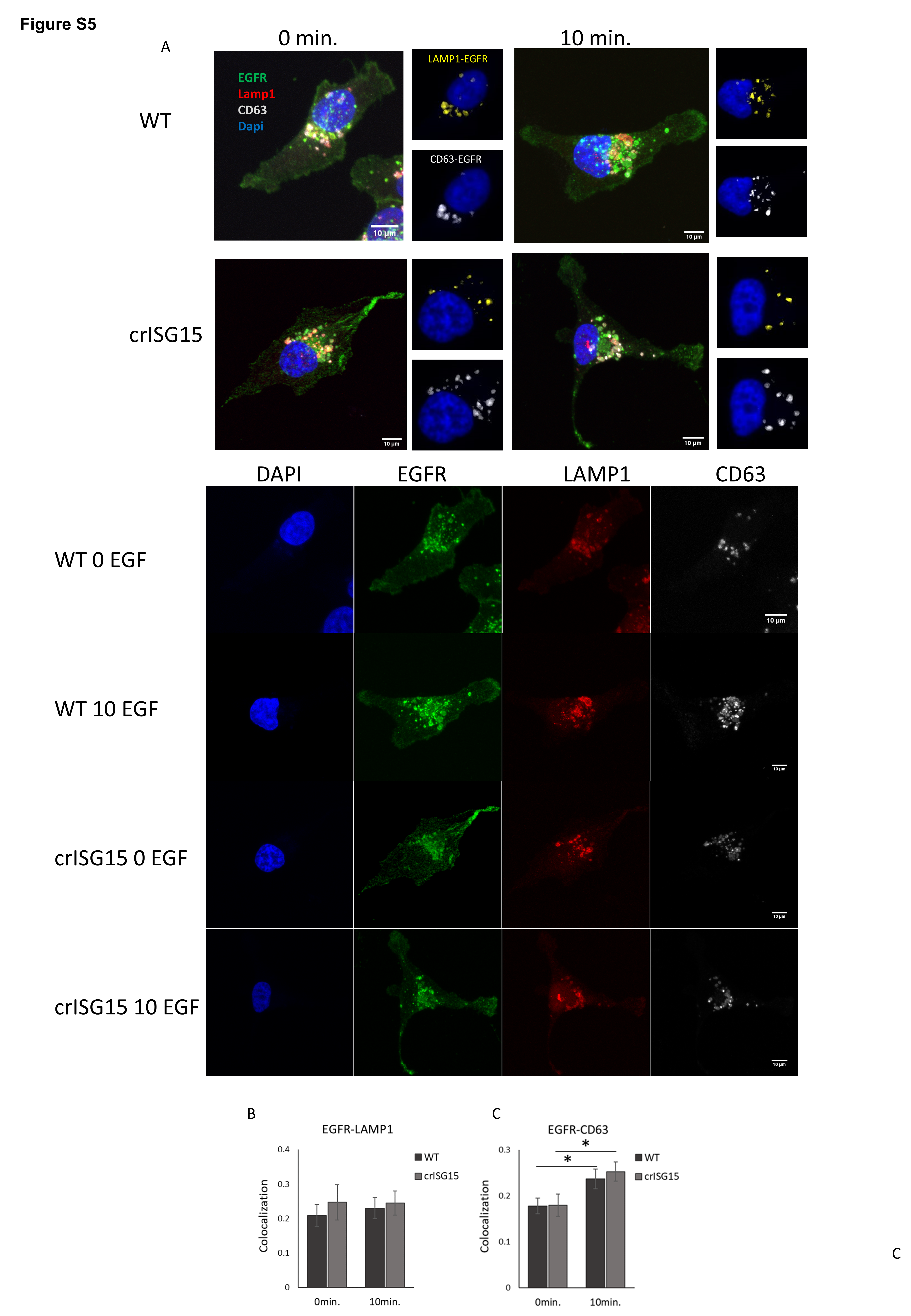

Supplement: Supplementary file 6 — Figure S5 [file 41388_2021_2017_MOESM6_ESM.tif]

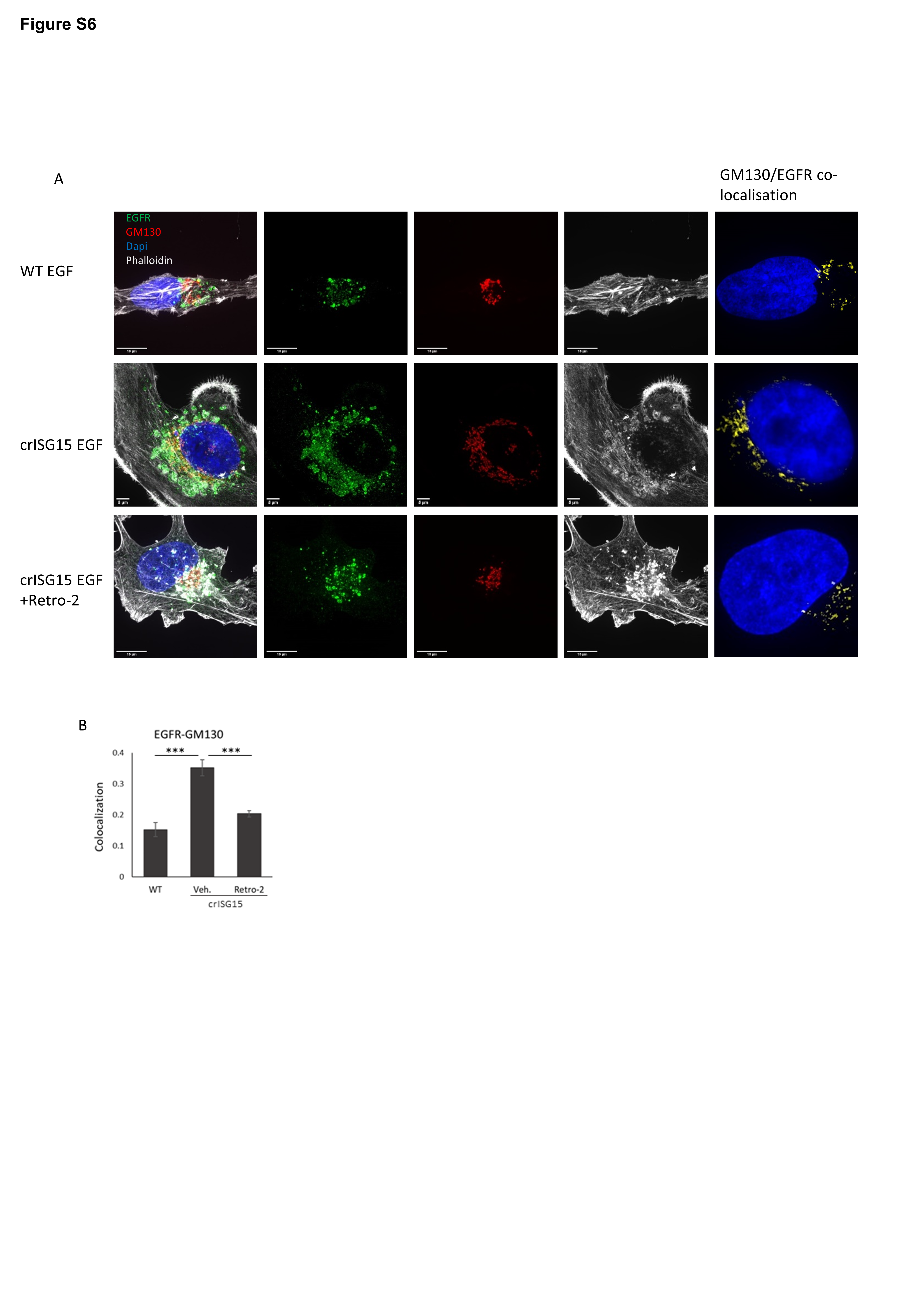

Supplement: Supplementary file 7 — Figure S6 [file 41388_2021_2017_MOESM7_ESM.tif]

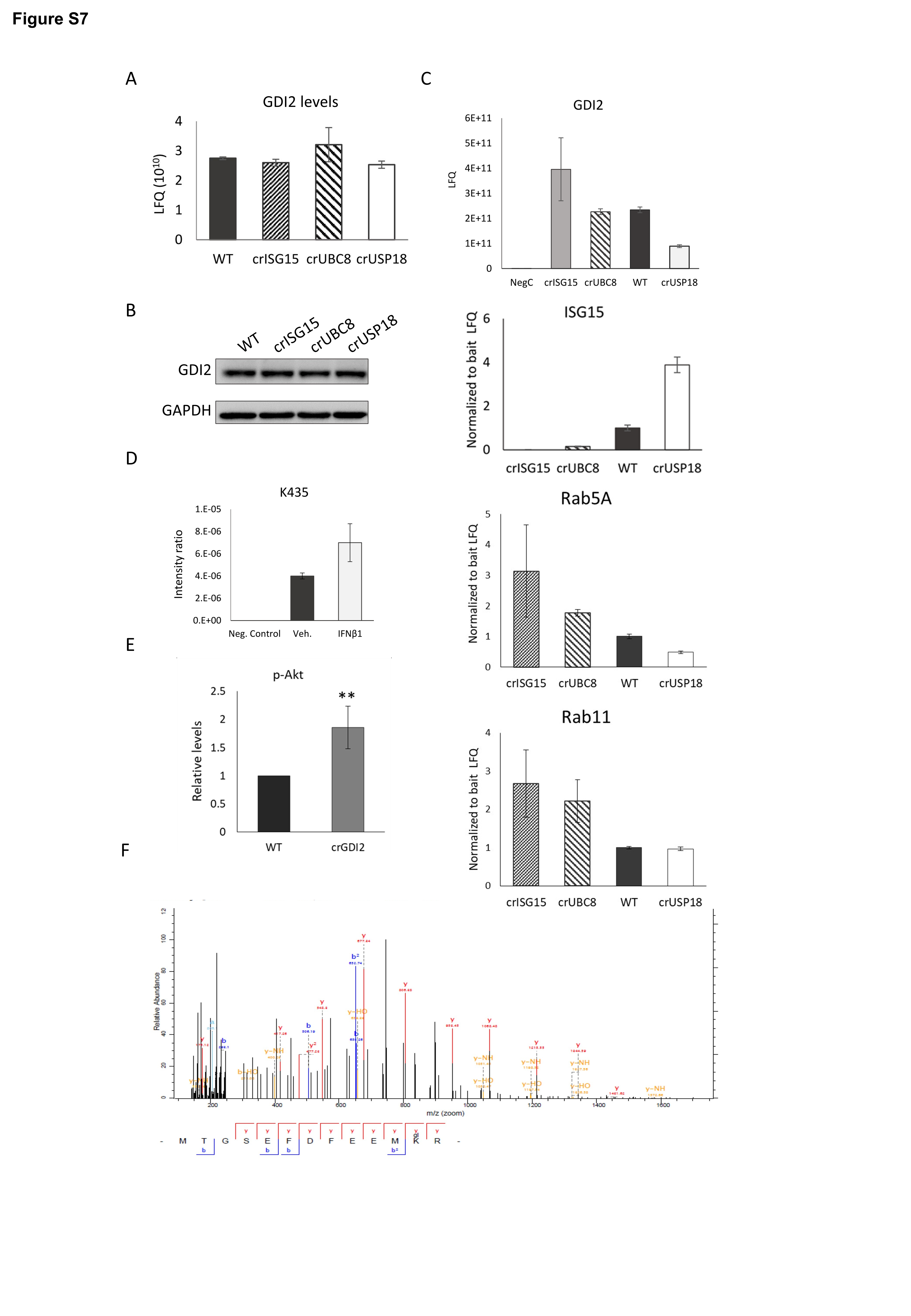

Supplement: Supplementary file 8 — Figure S7 [file 41388_2021_2017_MOESM8_ESM.tif]

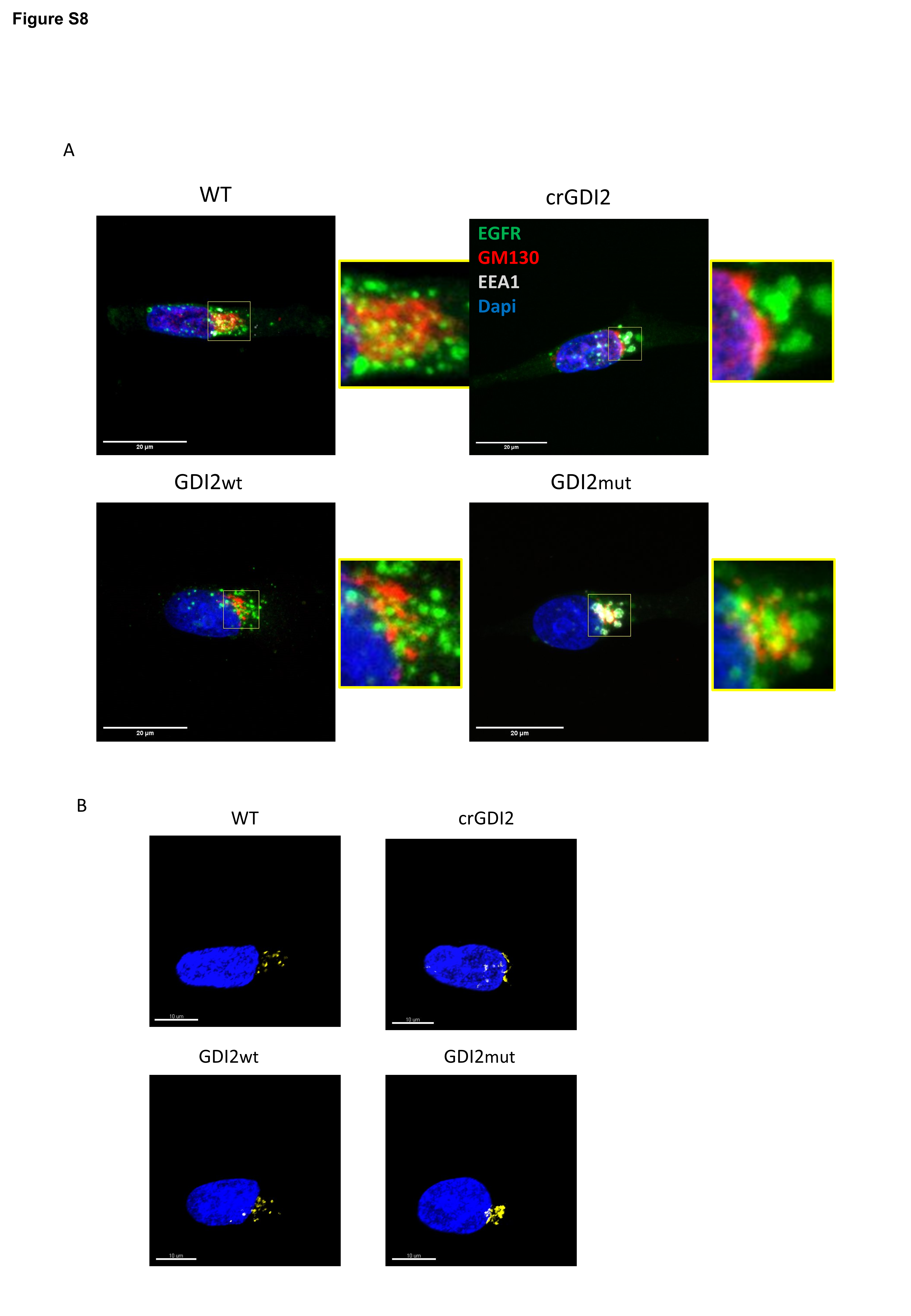

Supplement: Supplementary file 9 — Figure S8 [file 41388_2021_2017_MOESM9_ESM.tif]
